# Physics-constrained and geometry-aware deep reconstruction for limited-angle CBCT

**DOI:** 10.1088/1361-6560/aeb59b

**Published:** 2026-11-03

**Authors:** Mingzhe Hu, Shaoyan Pan, Yuan Gao, Huiqiao Xie, Jill Remick, Chih-Wei Chang, Justin Roper, Zhen Tian, Xiaofeng Yang

**Affiliations:** 1 Department of Radiation Oncology and Winship Cancer Institute, Emory University, Atlanta, GA, United States of America; 2 Department of Radiation & Cellular Oncology, University of Chicago, Chicago, IL, United States of America

**Keywords:** limited-angle CBCT, cone-beam CT, image-guided radiotherapy, diffusion model, geometry-aware reconstruction, physics-constrained deep learning

## Abstract

*Objective.* Limited-angle cone-beam computed tomography (CBCT) is attractive for image-guided radiotherapy and imaging-guided procedures when full-arc acquisition is impractical, but the incomplete angular sampling leads to severe streaking, shading, and structural distortion. This study aimed to develop a physics-constrained and geometry-aware deep reconstruction framework for recovering anatomically faithful volumetric CBCT from short-arc limited-angle projections. *Approach.* We propose a physics-constrained and geometry-aware network (PCGA-Net), a dual-domain reconstruction framework that combines projection completion, analytic geometric reconstruction, and image-domain refinement within a unified cross-domain design. Specifically, a projection completion module first estimates missing-angle information from the measured limited-angle projections. A geometry-aware reconstruction module then maps the completed projections into image space using differentiable cone-beam forward and backward-projection operators. Finally, a volumetric refinement module suppresses residual artifacts and restores anatomical detail under geometry-guided cross-domain consistency supervision. The framework was evaluated on three clinically distinct cohorts: gynecologic (GYN) high-dose-rate (HDR) brachytherapy, abdominal liver four-dimensional computed tomography (4DCT), and head-and-neck (H&N) CBCT. *Main results.* Across all three cohorts, fully adapted PCGA-Net consistently outperformed conventional limited-angle Feldkamp–Davis–Kress (FDK) reconstruction and the compared learning-based baselines, a conditional generative adversarial network (cGAN) and PatRecon-Net, in both qualitative and quantitative evaluation under matched patient-specific adaptation conditions. On the GYN HDR brachytherapy cohort, PCGA-Net achieved a mean absolute error (MAE) of 34.47 ± 6.28 Hounsfield units (HU), a structural similarity index measure (SSIM) of 0.87 ± 0.04, and a peak signal-to-noise ratio (PSNR) of 35.79 ± 0.95 dB, compared with 178.63 ± 31.58 HU, 0.51 ± 0.03, and 18.97 ± 1.31 dB for FDK. On the abdominal liver 4DCT cohort, PCGA-Net achieved 39.27 ± 6.10 HU MAE, 0.82 ± 0.03 SSIM, and 36.85 ± 1.16 dB PSNR, versus 195.21 ± 40.08 HU, 0.50 ± 0.06, and 18.50 ± 1.50 dB for FDK. On the H&N CBCT cohort, PCGA-Net achieved 28.42 ± 4.88 HU MAE, 0.91 ± 0.05 SSIM, and 38.52 ± 0.95 dB PSNR, compared with 165.30 ± 25.40 HU, 0.61 ± 0.05, and 21.13 ± 1.14 dB for FDK. In all three cohorts, PCGA-Net also exceeded cGAN and PatRecon-Net across all evaluated metrics. *Significance.* These results indicate that jointly enforcing projection-domain completion, analytic imaging geometry, and image-domain refinement is an effective strategy for limited-angle CBCT reconstruction. PCGA-Net provides a robust short-arc reconstruction framework across multiple anatomical sites and clinical workflows, supporting its potential utility in constrained image-guided therapeutic and procedural settings.

## 1. Introduction

In contemporary image-guided therapy, the practical question is often not whether volumetric x-ray imaging is desirable, but whether a full cone-beam orbit can be executed without compromising the treatment or procedure itself. In external beam radiotherapy, cone-beam computed tomography (CBCT) is routinely needed for setup verification, anatomy-of-the-day assessment, and motion-aware image guidance; in brachytherapy and other intracavitary or interstitial procedures, volumetric imaging is equally valuable for applicator-based target localization and peri-procedural verification; and in CBCT-guided interventional or surgical workflows, three-dimensional visualization must often be obtained without disrupting instrument placement or sterile workflow (Jensen *et al* 2019). Yet across these settings, full-arc acquisition is frequently constrained by prolonged gantry rotation, intrafraction anatomical change, patient discomfort or limited tolerance, gantry–couch clearance, immobilization hardware, anesthesia equipment, sterile draping, and the physical footprint of needles, applicators, or other procedural devices (Hueso-González *et al* 2020, Lei *et al* 2024). Under such conditions, continuous short-arc acquisition is not merely a reduced version of standard CBCT, but a more realistic and often safer imaging mode that better fits crowded treatment environments and time-sensitive procedural workflows while remaining compatible with existing onboard or C-arm systems.

However, the clinical appeal of short-arc acquisition is inseparable from a substantially harder reconstruction problem. When the gantry traverses only a limited angular range, the resulting projection set no longer provides the complete directional information assumed by conventional cone-beam reconstruction, and the missing angular coverage propagates into the image as structured artifact rather than simple noise (Feldkamp 1984, Barutcu *et al* 2021). In practice, this incomplete angular support leads to characteristic degradation patterns, including streaking, shading, blurring, and local geometric distortion, which can obscure soft-tissue boundaries and compromise volumetric fidelity (Bhadra *et al* 2021). These errors are especially problematic in anatomy-dependent image guidance, where subtle boundary shifts or low-contrast soft-tissue changes may carry direct clinical relevance. Therefore, limited-angle CBCT should not be viewed as simply a lower-dose or faster variant of standard acquisition, but as an intrinsically more ill-conditioned reconstruction setting in which incomplete projection information must be compensated without violating the physical consistency of the measured data.

Within this broader landscape of geometry-constrained volumetric reconstruction, our prior studies have examined two clinically meaningful but highly specific acquisition regimes: single-view reconstruction and orthogonal-view reconstruction. The single-view setting represents the most extreme discrete geometry, in which a three-dimensional CBCT volume is inferred from one arbitrary projection under strong patient-specific conditioning (Pan *et al* 2025). However, many real clinical workflows are not governed by an isolated single view or a strictly biplanar snapshot pair. Instead, they more often yield a continuous short arc of projections, which preserves local angular continuity while still leaving a substantial missing-angle burden. In this sense, continuous limited-angle acquisition constitutes a more general and clinically prevalent geometric constraint, positioned between highly discrete special cases and conventional full-arc CBCT, and therefore requires a reconstruction framework designed specifically for its distinct balance of continuity, incompleteness, and geometric fidelity.

A substantial body of prior work has attempted to mitigate limited-angle degradation, but the remaining gap is not solely one of image quality; it is the difficulty of jointly preserving artifact suppression, geometric fidelity, and anatomical realism under incomplete angular sampling. On the model-based side, Shi and Qu (2022) developed an ultra-limited-angle reconstruction algorithm based on reweighting and edge-preserving regularization, Jia *et al* (2022) proposed a superiorization-inspired unrolled simultaneous algebraic reconstruction technique framework with learned perturbations for sparse-view and limited-angle computed tomography (CT), and Lee and Baek (2024) further explored iterative reconstruction with implicit neural representations; these approaches benefit from explicit use of the forward model, yet they still rely on iterative optimization or fixed update structures whose performance remains sensitive to the severity of angular truncation and the acquisition setting (Ritschl *et al* 2011, Adler and Öktem 2018, Huang *et al*, 2020, Wu *et al* 2021). In parallel, image-restoration strategies have shown that post-reconstruction learning can visibly reduce wedge artifacts: Wang *et al* (2020) combined a Feldkamp–Davis–Kress (FDK)-based neural network with a U-Net for limited-angle CT, Hu *et al* (2023) proposed SEA-Net for limited-angle CBCT reconstruction from clinical projection data, Zhang *et al* (2023) developed TIME-Net for limited-angle artifact removal in dual-energy CBCT, and more recently hybrid U-Net/transformer designs have been reported for limited-angle cardiac CT. However, these methods remain largely image-centric by construction, and therefore may improve visual appearance without fully resolving the projection inconsistency induced by missing angles. Generative reconstruction has opened a further direction. Liu *et al* (2023) introduced DOLCE to combine diffusion sampling with an explicit physical measurement model for limited-angle CT, Zhang *et al* (2024) proposed a wavelet-inspired multi-channel score-based model to better address directional artifact structure, Wang *et al* (2024) developed a time-reversion fast-sampling score-based model to reduce the sampling burden of score-based reconstruction, and a recent physics-informed score-based diffusion model incorporated primal-dual hybrid gradient-based data consistency for limited-angle cardiac CT Han *et al* (2024). These studies indicate that generative priors can recover sharper and more realistic structures, but they also underscore that, under incomplete projections, anatomical plausibility alone is insufficient unless the reconstruction is tightly anchored to scanner geometry and measured data. Accordingly, continuous short-arc CBCT still lacks a framework that can simultaneously reconcile incomplete-projection compensation, explicit geometric consistency, and anatomically faithful volumetric reconstruction.

To address these challenges, we propose a physics-constrained and geometry-aware network (PCGA-Net) framework that treats continuous short-arc CBCT reconstruction as a coordinated projection-to-image inference problem rather than a single-domain correction task. The central idea is that limited-angle reconstruction requires simultaneous recovery of missing projection information and refinement of the corresponding volumetric anatomy under the true cone-beam acquisition geometry. Accordingly, PCGA-Net integrates three tightly coupled components within one unified pipeline. A projection completion module (PCM) first completes the unmeasured views from the available short-arc projections, providing a data-driven estimate of the missing angular information. The completed projection set is then passed through a geometry-aware reconstruction module (GARM), implemented with analytic cone-beam forward- and back-projectors, to map projection-domain inference into image space while preserving scanner-specific geometric fidelity (Feldkamp 1984, Ho *et al* 2020, Kim and Champley 2023). On this basis, a volumetric refinement module (VRM) further suppresses residual limited-angle artifacts and restores fine anatomical detail in the reconstructed volume. Finally, a projection-image-projection cycle-domain supervision strategy enforces consistency between synthesized projections, reconstructed volumes, and the originally measured data, thereby constraining the generative process with explicit physical geometry. Through this dual-domain, geometry-integrated design, PCGA-Net extends our prior geometry-aware reconstruction framework to the clinically important setting of continuous limited-angle CBCT and is intended to provide artifact-reduced, anatomically faithful volumetric images from short-arc acquisitions.

Building upon these considerations, the contributions of this work are as follows:

1. As a clinically important and geometrically constrained imaging problem, we try to formulate the continuous limited-angle CBCT reconstruction as a unified framework, in which a short acquisition arc provides locally continuous projections but still leaves substantial missing-angle information to be recovered. This setting extends beyond highly discrete sparse-angle acquisition scenarios and better reflects a broad range of practical image-guided therapeutic and procedural workflows.
2. We propose PCGA-Net, a geometry-integrated dual-domain framework that jointly combines missing-angle projection completion, analytic cone-beam geometry transformation, and volumetric refinement within a unified projection-image-projection cycle. By coordinating inference across projection and image domains, the proposed framework is designed to address incomplete angular sampling more effectively than single-domain reconstruction strategies.
3. In contrast to conventional image-domain artifact reduction methods, fixed iterative reconstruction schemes, and purely generative approaches, PCGA-Net explicitly incorporates scanner-specific geometric consistency into the reconstruction process. This design enables the model to improve artifact suppression and anatomical detail restoration while reducing the risk of losing image fidelity that are insufficiently supported by the measured data.
4. Relative to our previous preprint version (Gao *et al* 2025), the present study substantially expands the validation scope beyond a single pelvic application and evaluates the proposed framework across multiple anatomical sites with different structural complexity and workflow characteristics, including pelvis, abdomen, and head-and-neck (H&N) cases. This broader evaluation strengthens the evidence for the cross-site robustness under site-specific population pretraining and the specified longitudinal patient-adaptation protocol, and clinical relevance of PCGA-Net in diverse short-arc CBCT settings.

## 2. Methodology

We propose PCGA-Net, a dual-domain generative framework for reconstructing high-fidelity CBCT volumes from continuous limited-angle projections. The design is motivated by the denoising diffusion probabilistic model (DDPM), which formulates generation as a reverse denoising process from Gaussian noise, and by the long-established use of analytic cone-beam reconstruction operators, such as the FDK algorithm, for geometry-based volumetric recovery. Rather than treating short-arc CBCT as a purely image-domain restoration problem, PCGA-Net formulates reconstruction as a coordinated projection-to-volume inference task under explicit cone-beam acquisition geometry. Consistent with the current PCGA-Net framework, the pipeline couples three differentiable components: a PCM to estimate the missing views from the measured short-arc projections, a GARM implemented with analytic cone-beam forward- and backward-projectors to map projection-domain inference into image space, and a VRM to further suppress residual limited-angle artifacts and recover anatomically relevant detail in the reconstructed volume (Feldkamp 1984, Kim and Champley 2023). This projection–geometry–image formulation also follows the broader logic established in prior geometry-integrated CBCT reconstruction work from our group, in which dual-domain generative modeling and explicit scanner geometry were used to strengthen data fidelity under severely restricted acquisition conditions (Pan *et al* 2024, Pan *et al* 2025). Overall, PCGA-Net is designed to integrate complementary priors from projection and image spaces while constraining the reconstruction with analytic imaging physics, thereby extending geometry-aware diffusion-based CBCT reconstruction to the clinically important setting of continuous short-arc limited-angle acquisition.

### 2.1. Clinical cohort

To evaluate PCGA-Net under clinically distinct but complementary imaging conditions, we used three datasets spanning abdominal liver four-dimensional CT (4DCT), H&N CBCT, and gynecologic (GYN) high-dose-rate (HDR) brachytherapy CT. This combination was selected to probe limited-angle reconstruction across markedly different workflow demands, motion patterns, and structural compositions rather than within a single anatomical niche. The abdominal liver 4DCT cohort represents an upper-abdominal radiotherapy setting in which respiratory motion is a dominant source of anatomical variability, and 4DCT is routinely used to characterize motion envelopes and support motion-aware target definition in liver radiotherapy. The H&N CBCT cohort represents a daily image-guided and adaptive radiotherapy scenario in which on-board CBCT is clinically relevant for setup verification, surveillance of treatment-induced anatomical change, and adaptive decision-making. The GYN HDR brachytherapy CT cohort represents a procedure-oriented pelvic setting in which volumetric imaging is required for applicator or needle verification, target localization, and peri-procedural assessment, often under severe workspace and clearance constraints. Consistent with prior geometry-integrated CBCT reconstruction studies from our group, the liver 4DCT and GYN HDR brachytherapy CT datasets were used to generate simulated cone-beam projections under known acquisition geometry, whereas reconstructed clinical on-board CBCT volumes were used as source volumes for matched forward-projection simulation in the H&N cohort.

For CBCT reconstruction research, CT-based forward simulation provides a cleaner reference standard because the underlying CT volumes are anatomically resolved and quantitatively stable, allowing the generated projections and reconstructed volumes to remain exactly paired under a prescribed cone-beam geometry. In contrast, real CBCT images already contain scatter contamination, noise, truncation effects, and system-dependent artifacts, which can obscure the anatomical ground truth and confound algorithmic evaluation. For this reason, CT- or 4DCT-based projection simulation is particularly suitable for the liver and GYN cohorts, where the goal is to assess how accurately the model recovers volumetric anatomy from incomplete angular measurements under controlled physical conditions, while the H&N CBCT cohort complements this design by evaluating controlled re-projection/reconstruction performance on clinically derived on-board CBCT anatomy where matched planning CT was not uniformly available.

All projection-domain inputs in this study were generated under a prescribed cone-beam geometry. For the GYN and liver cohorts, CT or 4DCT volumes were forward-projected to generate paired full-view and limited-angle data. For the H&N cohort, reconstructed clinical on-board CBCT volumes served as the source volumes and reference targets. Because vendor raw detector projections were not available, each reconstructed H&N CBCT volume was forward-projected with LEAP-CT to generate a synthetic 360-view projection stack, from which the fixed 90-view short arc was retained as the model input. Therefore, the H&N experiment uses clinically acquired reconstructed CBCT anatomy but simulated projection measurements; it is not an evaluation on original raw detector data.

These three datasets also provide a test for model robustness. Liver 4DCT emphasizes low-contrast soft tissue under respiration-driven deformation; H&N CBCT emphasizes complex bone-air-soft-tissue interfaces, immobilization-related geometry, and fine structural detail; and GYN HDR brachytherapy CT emphasizes crowded pelvic anatomy together with metallic applicators or interstitial needles that complicate limited-angle reconstruction. Evaluating all three therefore allows us to assess whether PCGA-Net remains effective across dynamic abdominal anatomy, anatomically intricate H&N image guidance, and device-heavy interventional radiotherapy, which is more informative than validation in a single pelvic-only context. This broader multi-site design is aligned with the clinical motivation of the present study, namely, to develop a short-arc CBCT reconstruction framework that is robust to anatomical heterogeneity, workflow variability, and challenging imaging conditions encountered in real image-guided therapeutic procedures.

### 2.2. PCGA-Net framework

For clarity, the principal notation used in the following PCGA-Net framework is summarized in table 1 before the individual components are introduced.

**Table 1.** Summary of notation used in the proposed PCGA-Net framework. The variables are grouped into projection completion module (PCM) variables, volumetric refinement module (VRM) variables, and geometry-aware reconstruction module (GARM)/cross-domain supervision variables.

Symbol	Definition	Symbol	Definition

PCM variables			
$F{P_0^{\mathrm{real}}}$	Clean ground-truth full-view projection target.	$F{P_t^{\mathrm{real}}}$	Noisy training sample obtained by perturbing $F{P_0^{\mathrm{real}}}$ at timestep t.
${P_0^{\mathrm{syn}}}$	Clean synthesized projection obtained at the end of the reverse diffusion process.	${P_0^{\mathrm{real}}}$	Measured 90-view limited-angle projection used only as the conditional input.
${\bar{\alpha}_t}$	Cumulative noise schedule coefficient at timestep t, defined as ${\bar{\alpha}_t} = \prod \limits_{s = 1}^{t}{\alpha_s}$.	${\alpha_t}$	Noise schedule scalar at timestep t.
${\beta_t}$	Noise schedule coefficient at timestep t, where ${\alpha_t} = 1-{\beta_t}$.	ϵ	Gaussian noise sampled from $\mathrm{N}\left( 0,I \right)$ in the forward diffusion process.
${\epsilon_\theta}\left( {P_t^{\mathrm{syn}}},t,{P_0^{\mathrm{real}}} \right)$	Noise prediction in the projection domain estimated by the PCM.	${\sigma_t}$	Variance or standard deviation of the reverse diffusion step at timestep t.
z	Gaussian random variable sampled from $\mathrm{N}\left( 0,I \right)$ in the reverse diffusion process.	t	Diffusion timestep index, where $t \in \left[ 1,T \right]$.
T	Total number of diffusion steps.	${P_s^{\mathrm{syn}}}$	Projection-state variable along the learned reverse trajectory.
${P_s^{\mathrm{det}}}$	Deterministic full-view projection generated by the PCM at timestep s.	${P_s^{\mathrm{sto}}}$	Stochastic projection sample generated by the PCM at timestep s.

VRM variables			

Symbol	Definition	Symbol	Definition

${I_0^{\mathrm{real}}}$	Clean ground-truth reference volume.	${I_t^{\mathrm{real}}}$	Noisy training sample obtained by perturbing ${I_0^{\mathrm{real}}}$ at timestep t.
${I_0^{\mathrm{syn}}}$	Final clean CT output synthesized by the VRM.	${I_s^{\mathrm{sto}}}$	Stochastic image sample generated by the VRM at timestep s.
${\mathrm{L}_\theta^{\mathrm{I}\mathrm{,}\mu}}$	Mean prediction loss in the VRM.	${\mathrm{L}_\theta^{\mathrm{I}\mathrm{,}\Sigma}}$	Variance prediction loss in the VRM.
${I_s^{\mathrm{syn}}}$	Image-state variable along the learned reverse trajectory.	${I_s^{\mathrm{det}}}$	Deterministic image output generated by the VRM at timestep s.

GARM and cross-domain supervision variables			

Symbol	Definition	Symbol	Definition

$\mathrm{R}\left( \cdot \right)$	Analytic reconstruction operator in the GARM that maps synthesized projections to an intermediate volumetric reconstruction.	$\mathrm{F}\left( \cdot \right)$	Analytic forward projection operator in the GARM that maps image-domain outputs back to projection space.
${I^{\mathrm{rec}}}$	GARM-derived intermediate reconstruction used as the VRM condition.	${I^{\mathrm{real-rec}}}$	Ground-truth CT volume reconstructed from $F{P^{\mathrm{real}}}$ using the same GARM.
${I_0^{\mathrm{cycle}}}$	Final CT volume generated in the cycle-supervision branch, representing the synthesized CT under cycle guidance.	${I_t^{\mathrm{cycle}}}$	Noisy CT volume at timestep t reconstructed from forward-projected synthetic projections through the GARM during cycle supervision.
${P_s^{\mathrm{rec}}}$	GARM-derived projection-domain reference used to guide the PCM during reverse generation.	${\epsilon_\theta}\left( {I_t^{\mathrm{cycle}}},t,{I^{\mathrm{rec}}} \right)$	Noise prediction in the image domain estimated by the VRM under cycle supervision.
${\mathrm{L}_{\mathrm{CT-rec}}}$	GARM-based reconstruction supervision loss between ${I^{\mathrm{real-rec}}}$ and ${I^{\mathrm{rec}}}$.	${\mathrm{L}_{\mathrm{CT-cycle}}}$	Cycle-supervision loss between ${I_0^{\mathrm{real}}}$ and ${I_0^{\mathrm{cycle}}}$.
${\mathrm{L}_{\mathrm{CT}}}$	Total image-side objective combining diffusion, reconstruction, and cycle-supervision losses.	${\gamma_1},{\gamma_2},{\gamma_3}$	Empirical weighting coefficients used to balance the components of ${\mathrm{L}_{\mathrm{CT}}}$.
S	Total number of reverse generation timesteps used during the application process.	${X_s}$	Generic noisy state at reverse generation timestep s, representing either the projection or image variable.
${\mu_s^\theta}\left( \cdot \right)$	Mean term predicted by the diffusion model at reverse timestep s during stochastic sampling.	${\Sigma_s^\theta}\left( \cdot \right)$	Variance term predicted by the diffusion model at reverse timestep s during stochastic sampling.

#### 2.2.1. PCM

The PCM is designed to recover a complete projection representation from the measured limited-angle input and serves as the first generative stage of the PCGA-Net framework. Its role is not to reconstruct the CBCT volume directly, but to infer the missing angular information in projection space so that the downstream GARM can operate on a physically more complete projection set. In this way, the projection-domain task is formulated as a conditional inpainting problem under limited-angle acquisition, where the model learns to synthesize full-view projections from incomplete measured views while remaining guided by the available short-arc data. This design follows the same dual-domain logic established in our previous geometry-integrated CBCT frameworks, while adapting it here to the continuous short-arc setting of limited-angle CBCT. As illustrated in figure 1(a), the PCM takes the measured limited-angle projections as conditional input and predicts the corresponding full-view projection representation for subsequent geometric reconstruction.

**Figure 1.**
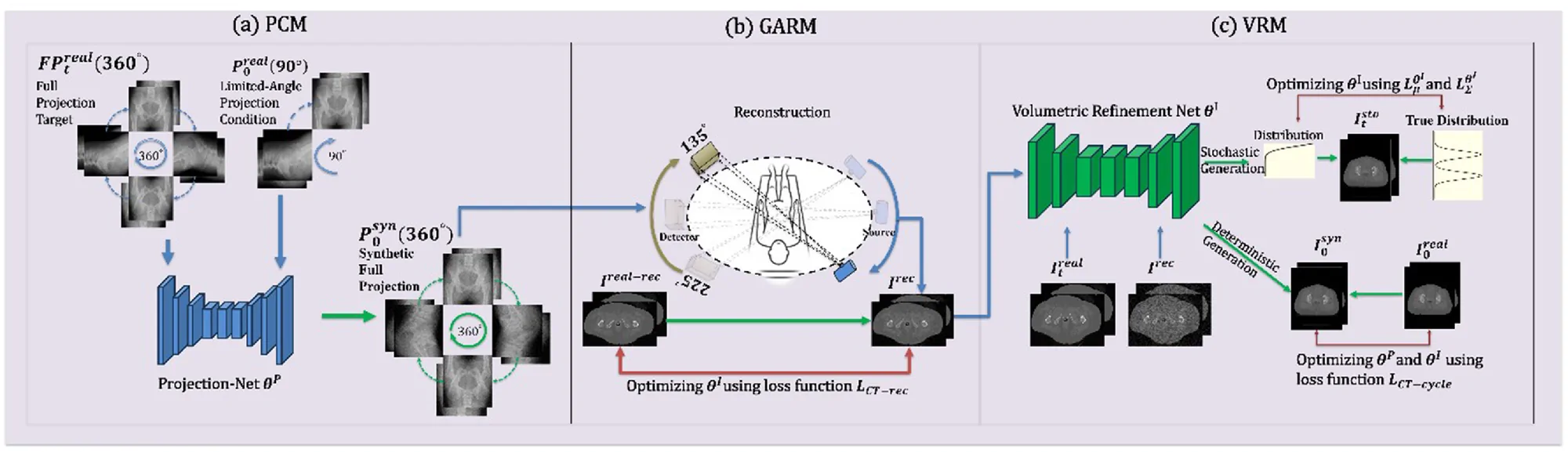
Supervised training framework of PCGA-Net, comprising the Projection Completion Module (PCM), Geometry-Aware Reconstruction Module (GARM), and Volumetric Refinement Module (VRM). Panels are read from left to right as (a) PCM, (b) GARM, and (c) VRM. The schematic summarizes module-level computational and supervision dependencies; individual forward-noising operations, noise predictions, and reverse-diffusion updates are not separately drawn. (a) PCM. The noisy-state input is $F{P_t^{\mathrm{real}}}$, obtained by perturbing the clean full-view target $F{P_0^{\mathrm{real}}}$ according to equations (1) and (2). The conditioning input is the fixed 90-view limited-angle projection ${P_0^{\mathrm{real}}}$, labeled ${P_t^{\mathrm{real}}}$ in the schematic; this condition is not subjected to forward diffusion. Given the noisy state, timestep t, and condition, the projection-domain denoiser predicts ${\epsilon_\theta}\left( F{P_t^{\mathrm{real}}},t,{P_0^{\mathrm{real}}} \right)$. The displayed synthesized full-view projection ${P_0^{\mathrm{syn}}}$ represents the clean endpoint of the learned reverse-diffusion process in equation (3), rather than the direct output of a single denoiser evaluation. (b) GARM. The geometry-informed intermediate volume ${I^{\mathrm{rec}}}$ is reconstructed from the synthesized full-view projection ${P_0^{\mathrm{syn}}}$ using a differentiable analytic cone-beam reconstruction operator. Reconstruction supervision is applied under the same acquisition geometry. Specifically, ${I^{\mathrm{rec}}}\mathrm{=}\mathrm{R}\left( {P_0^{\mathrm{syn}}} \right)$ is compared with ${I^{\mathrm{real-rec}}}\mathrm{=}\mathrm{R}\left( F{P_0^{\mathrm{real}}} \right)$ through ${\mathrm{L}_{\mathrm{CT-rec}}}$. (c) VRM. The noisy-state input ${I_t^{\mathrm{real}}}$ is obtained from the clean reference volume ${I_0^{\mathrm{real}}}$ using equations (5) and (6), and ${I^{\mathrm{rec}}}$ provides the geometric condition. The volumetric denoiser predicts ${\epsilon_\theta}\left( {I_t^{\mathrm{real}}},t,{I^{\mathrm{rec}}} \right)$; the reverse-diffusion process produces the displayed synthesized volume ${I_0^{\mathrm{syn}}}$. The distribution symbols summarize stochastic sampling and the associated reverse-process distribution losses. The lower supervision branch compares the cycle-guided image output with the reference volume through ${L_{{\text{CT - cycle}}}}$. Blue arrows indicate transmission of network inputs and geometry-derived quantities; connections from the limited-angle projection to PCM and from ${I^{\mathrm{rec}}}$ to VRM are conditioning pathways. Green arrows depict generation branches and associated reference comparisons. Red/orange brackets identify the quantities involved in the labeled optimization losses. Here, $t \in \{ 1, \ldots ,{\mathrm{T}}\}$ is a training diffusion timestep and T=1000; the labels $9{0^\circ}$ and $36{0^\circ}$ indicate angular coverage.

Let $F{P_0^{\mathrm{real}}}$ denote the clean ground-truth full-view projection target and ${P_0^{\mathrm{real}}}$ denote the acquired limited-angle projection set used as the conditional input. The PCM learns the reverse diffusion process that maps Gaussian noise to the clean synthesized projection ${P_0^{\mathrm{syn}}}$, conditioned on ${P_0^{\mathrm{real}}}$.

During training, Gaussian noise is added to $F{P_0^{\mathrm{real}}}$ through the fixed forward diffusion process. At diffusion timestep t, the noised training sample $F{P_t^{\mathrm{real}}}$ is obtained from $F{P_0^{\mathrm{real}}}$ according to equation (1).

\begin{equation*}q\left( F{P_t^{\mathrm{real}}}\mid F{P_0^{\mathrm{real}}} \right)\mathrm{=N}\left( F{P_t^{\mathrm{real}}};\sqrt{{\bar{\alpha}_t}} F{P_0^{\mathrm{real}}},\left( 1-{\bar{\alpha}_t} \right)\mathbf{I} \right),\end{equation*}

where ${\bar{\alpha}_t} = \prod \nolimits_{s = 1}^{t}{\alpha_s}$, ${\alpha_t} = 1-{\beta_t}$, and ${\beta_t}$ is the predefined noise schedule. Equivalently, the noisy sample can be written in reparameterized form as:

\begin{equation*}F{P_t^{\mathrm{real}}} = \sqrt{{\bar{\alpha}_t}} F{P_0^{\mathrm{real}}}+\sqrt{1-{\bar{\alpha}_t}} \epsilon ,\epsilon \sim \mathrm{N}\left( 0,I \right).\end{equation*}

This formulation enables direct sampling of the noisy projection at any timestep and provides the basis for training the reverse denoising network.

The reverse process is parameterized by a neural network ${\epsilon_\theta}$, which predicts the noise component in the noisy projection conditioned on the limited-angle measurement. Starting from Gaussian noise, the model iteratively denoises the latent variable and produces a clean full-view synthesized projection. The reverse update at timestep t is written as:

\begin{equation*}{P_{t-1}^{\mathrm{syn}}} = \frac 1{\sqrt{{\alpha_t}}}\left( {P_t^{\mathrm{syn}}}-\frac{1-{\alpha_t}}{\sqrt{1-{\bar{\alpha}_t}}}\;{\epsilon_\theta}\left( {P_t^{\mathrm{syn}}},t,{P_0^{\mathrm{real}}} \right) \right)+{\sigma_t}z,\;z \sim \mathrm{N}\left( 0,\boldsymbol{I} \right),\end{equation*}

where ${\sigma_t}$ denotes the variance term of the reverse diffusion step. Here, the conditioning variable ${P_0^{\mathrm{real}}}$ plays a central role: it anchors the denoising trajectory to the measured short-arc projections and constrains the model to synthesize projection content that is compatible with the observed anatomy and acquisition geometry. As a result, the PCM does not perform unconstrained projection generation; instead, it estimates the unmeasured angular content under explicit guidance from the available limited-angle measurements. During inference, this progressive denoising process corresponds to the projection-generation pathway shown in figures 2(c) and (a), where the predicted projections are iteratively refined and exchanged with the image-domain branch through the GARM.

**Figure 2.**
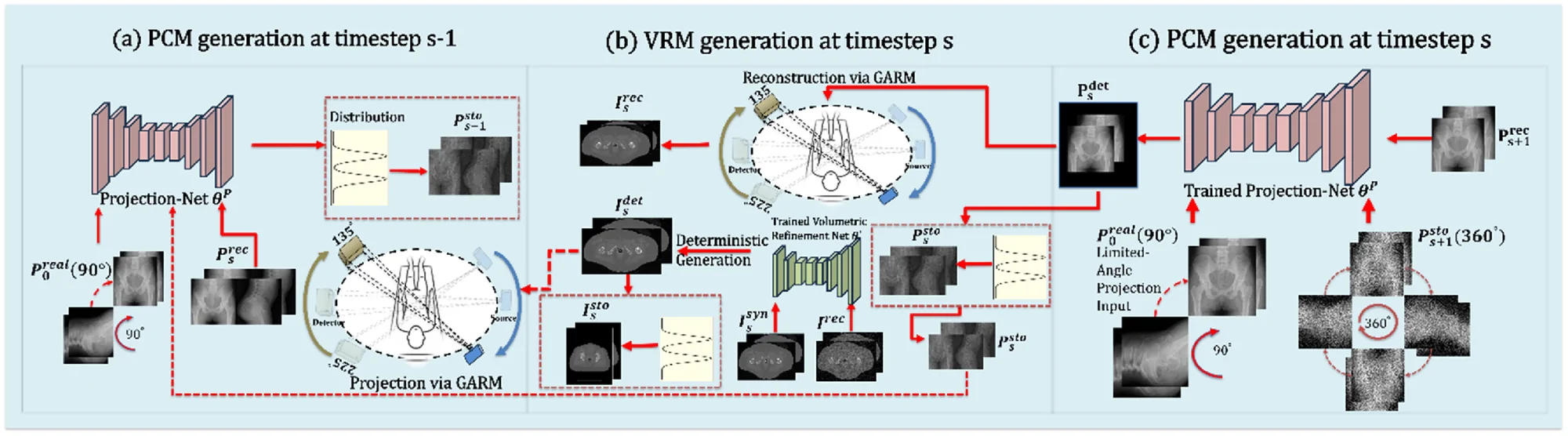
Geometry-coupled iterative inference of PCGA-Net. Read the panels from right to left as (c) → (b) → (a). The diagram illustrates a projection update, its associated volumetric update, and the feedback supplied to the next projection update. Projection- and image-domain states are initialized from Gaussian noise, and the reverse process uses S=50 sampling steps after timestep respacing. (c) PCM update. The noisy projection state ${P_{s+1}^{\mathrm{sto}}}$, measured limited-angle condition ${P_0^{\mathrm{real}}}$, and available geometry-derived projection reference ${P_{s+1}^{\mathrm{rec}}}$ enter the projection-domain update. The denoiser prediction parameterizes the reverse-diffusion operation, which produces the deterministic projection estimate ${P_s^{\mathrm{det}}}$ and stochastic state ${P_s^{\mathrm{sto}}}$. These displayed estimates are obtained through reverse updating; they are not direct noise-prediction outputs. (b) GARM and VRM update. The deterministic projection is analytically reconstructed as ${I_s^{\mathrm{rec}}}\mathrm{=R}\left( {P_s^{\mathrm{det}}} \right)$. This volume supplies the geometric condition for VRM, together with the current noisy image-domain state. The resulting reverse update produces ${I_s^{\mathrm{det}}}$ and ${I_s^{\mathrm{sto}}}$. The superscript ‘syn’ identifies a generated image-domain state and does not, by itself, imply that denoising is complete. (a) Geometric feedback. The deterministic volume is forward-projected as ${P_s^{\mathrm{rec}}}\mathrm{=F}\left( {I_s^{\mathrm{det}}} \right)$. This reference, the fixed limited-angle measurement, and the stochastic projection state ${P_s^{\mathrm{sto}}}$ are supplied to the subsequent PCM update, producing the state indexed by s−1. Deterministic estimates mediate geometric exchange between domains, while stochastic states advance the reverse trajectories. Repetition yields the final refined volume ${I_0^{\mathrm{syn}}}$. Red arrows show inference-time computation, conditioning, and state propagation; dashed enclosures group stochastic-sampling operations. No full-view ground-truth target, reference-volume target, or training loss is used in this inference diagram. The sampling index s is distinct from the training timestep t in figure 1.

The network is optimized by minimizing the discrepancy between the true Gaussian noise and the noise predicted by the model. The training objective for the PCM is given by:

\begin{equation*}{L_{\mathrm{PCM}}} = {\mathrm{E}_{{\mathrm{FP}_0^{\mathrm{real}}},{\mathrm{P}_0^{\mathrm{real}}},t,\epsilon}}\left[ \|\epsilon -{\epsilon_\theta}{\left( F{P_t^{\mathrm{real}}},t,{P_0^{\mathrm{real}}} \right)\|_2^2} \right].\end{equation*}

This objective encourages the model to learn the full reverse denoising trajectory from noisy projections back to clean full-view projections. Because the projection synthesis is performed in the acquisition domain, the resulting output preserves angular structure more naturally than image-domain post-processing alone and provides a stronger physical initialization for the subsequent reconstruction stage. The final clean output of this module, ${P_0^{\mathrm{syn}}}$, is then passed to the GARM for analytic cone-beam reconstruction into an intermediate volumetric representation, as summarized in figure 1(b).

A key practical feature of the PCM is that it explicitly separates the task of missing-angle recovery from the later task of volumetric artifact refinement. By first restoring the projection domain, the framework allows the missing angular information to be addressed where it arises physically, before image-domain refinement is performed. This ordering is consistent with the overall dual-domain philosophy of PCGA-Net and is particularly important in limited-angle CBCT, where the incompleteness originates in the projection measurements rather than in the reconstructed image alone. In this sense, the PCM provides the data-completion stage of the framework, while the downstream GARM and VRM convert that completed projection information into a geometry-consistent and anatomically refined CBCT volume.

#### 2.2.2. Geometry-aware reconstruction module (GARM)

The GARM serves as the physical bridge between the projection-domain synthesis and the image-domain refinement stages of PCGA-Net. Its purpose is to transform the completed projection set generated by the PCM into a volumetric representation that is explicitly consistent with the cone-beam acquisition geometry, rather than allowing the subsequent image reconstruction to rely solely on data-driven inference. In the proposed framework, the GARM is implemented as a differentiable analytic imaging operator composed of cone-beam forward projection and backward reconstruction modules. This design allows the synthesized projections to be mapped into image space through a physics-defined transformation, while also enabling reconstructed volumes to be re-projected back into projection space during cycle supervision. As illustrated in figure 1(b), the GARM receives the synthesized full-view projections from the PCM and produces an intermediate reconstruction, which then serves as the geometry-initialized input for the VRM. During inference, the same module also supports the reciprocal projection-image interaction shown in figure 2, enabling information exchange between the two domains throughout the generation process.

Formally, let ${P_0^{\mathrm{syn}}}$ denote the clean full-view projection synthesized by the PCM. The GARM applies an analytic reconstruction operator $\mathrm{R}\left( \cdot \right)$ to obtain an intermediate volume, ${I^{\mathrm{rec}}} =$ $\mathrm{R}\left( {P_0^{\mathrm{syn}}} \right).$

In our implementation, $\mathrm{R}\left( \cdot \right)$ follows the cone-beam reconstruction geometry used in the data generation process and provides a physically meaningful initialization in image space. Conversely, given an image-domain prediction such as ${I_0^{\mathrm{syn}}}$ or ${I_0^{\mathrm{cycle}}}$, the GARM can also apply the corresponding forward projection operator $\mathrm{F}\left( \cdot \right)$ to recover its projection-domain representation, ${P^{\mathrm{rec}}}\mathrm{=F}\left( {I_0^{\mathrm{syn}}} \right).$

These two operators jointly establish a bidirectional geometric mapping between projection space and image space. As a result, the GARM is not merely a reconstruction tool, but a differentiable physical constraint that links the two DDPM branches under the same scanner geometry. This geometric coupling is essential because the missing information originates in projection space, whereas the final anatomical fidelity must be realized in image space.

An important function of the GARM is that it provides a stricter conditioning signal for the VRM than a purely image-domain initialization would allow. The reconstructed intermediate volume ${I^{\mathrm{rec}}}$ is derived from the synthesized full-view projection through the known cone-beam geometry and therefore contains anatomically organized spatial information that is already aligned with the acquisition model. This geometry-initialized reconstruction is then used as a strong condition for downstream volumetric refinement, reducing the ambiguity that would otherwise arise if the VRM were asked to infer the CBCT volume directly from incomplete measurements alone. In the reverse direction, forward-projecting the image-domain output back to projection space provides a physically interpretable mechanism for checking whether the generated anatomy remains compatible with the synthesized and measured projections. This bidirectional role of the GARM is consistent with the geometry-integrated design established in our prior CBCT reconstruction frameworks, while here it is specifically adapted to the continuous short-arc limited-angle setting.

In practice, the GARM was implemented using LEAP-CT under a predefined cone-beam geometry matching the simulation setup. The source-to-detector distance and source-to-isocenter distance were set to 1500 mm and 1000 mm, respectively. The x-ray detector size was 768 × 1024 with a detector spacing of 0.78 mm, and projection views were uniformly defined over 0°-360°. The reconstruction diameter was 495 mm, with an in-plane pixel spacing of 0.9668 mm, slice thickness of 1.0 mm, and matrix size of 512 × 512. Under this configuration, the GARM provided matched forward- and backprojection operators for projection synthesis, intermediate reconstruction, and cycle-domain supervision, thereby ensuring that both domains were coupled through the same acquisition geometry rather than through loosely aligned learned mappings alone.

The full-view projection stack contained 360 uniformly spaced views, with angular positions ${\theta_k} = {k^\circ}$ for $k = 0, \ldots ,{\mathrm{359}}$. The limited-angle input retained 90 consecutive clockwise views defined by ${\theta_k} = {\left( 135+k \right)^\circ}$ for $k = 0, \ldots ,{\mathrm{89}}$. Thus, the discrete short-arc set covers the nominal half-open interval $[13{5^\circ},22{5^\circ})$ at spacing. This identical angular subset was used to generate the limited-angle inputs for PCGA-Net, FDK, a conditional generative adversarial network (cGAN), and PatRecon-Net.

Another key advantage of the GARM is differentiability. Because both the forward and backward operators are embedded into the training graph, supervision can propagate across domains during optimization. This allows the PCM to benefit from image-domain feedback and the VRM to benefit from projection-domain consistency, rather than training the two branches as isolated generators. In practice, the GARM therefore enables the cycle-domain supervision used in PCGA-Net by enforcing that synthesized projections, reconstructed intermediate volumes, and refined image outputs remain mutually compatible under a unified cone-beam imaging model. This is particularly important for limited-angle CBCT, where preserving geometric fidelity is as critical as reducing visible artifacts. By anchoring both generation branches to explicit scanner physics, the GARM helps ensure that the final reconstructed volume is not only visually improved but also physically plausible with respect to the underlying acquisition process.

#### 2.2.3. VRM

The VRM constitutes the volumetric refinement stage of PCGA-Net and is designed to improve the intermediate reconstruction generated by the GARM into a high-fidelity CBCT volume. Unlike the PCM, which operates in acquisition space to compensate for missing angular information, the VRM operates in image space and focuses on suppressing residual limited-angle artifacts, restoring anatomical structures, and improving volumetric fidelity. Its role is therefore not to infer the entire anatomy from scratch, but to refine the geometry-initialized reconstruction ${I^{\mathrm{rec}}}$ into a cleaner and anatomically more accurate image representation. This design is consistent with the dual-domain philosophy of the framework, in which missing-angle completion is first addressed in projection space and then followed by image-domain restoration under explicit geometric guidance. As illustrated in figure 1(c), the VRM receives the GARM-based intermediate reconstruction as a strong condition and progressively denoises the volumetric sample toward the target CBCT distribution. During inference, its generation process corresponds to the image-domain branch shown in figure 2(b), where the refined image estimate is iteratively updated and subsequently projected back through the GARM for cross-domain feedback.

Let ${I_0^{\mathrm{real}}}$ denote the clean ground-truth reference volume and ${I_0^{\mathrm{syn}}}$ denote the final clean volume synthesized by the VRM. During training, Gaussian noise is added to ${I_0^{\mathrm{real}}}$ through the fixed forward diffusion process. At timestep t, the noised training sample ${I_t^{\mathrm{real}}}$ is obtained from ${I_0^{\mathrm{real}}}$ according to equation (5), while ${I^{\mathrm{rec}}}$ is supplied as the GARM-derived conditioning input.

\begin{equation*}q\left( {I_t^{\mathrm{real}}}\mid{I_0^{\mathrm{real}}} \right)\mathrm{=N}\left( {I_t^{\mathrm{real}}};\sqrt{{\bar{\alpha}_t}} {I_0^{\mathrm{real}}},\left( 1-{\bar{\alpha}_t} \right)I \right),\end{equation*}

where ${\bar{\alpha}_t} = \prod \nolimits_{s = 1}^{t}{\alpha_s}$ is the cumulative noise coefficient and ${\alpha_t} = 1-{\beta_t}$ is determined by the predefined noise schedule. Equivalently, the noisy sample can be written as:

\begin{equation*}{I_t^{\mathrm{real}}} = \sqrt{{\bar{\alpha}_t}} {I_0^{\mathrm{real}}}+\sqrt{1-{\bar{\alpha}_t}}\,\,\epsilon ,\; \epsilon \sim \mathrm{N}\left( 0,I \right).\end{equation*}

This formulation allows the model to observe the target image at different noise levels and learn the reverse mapping from noisy volumetric samples to clean CBCT volumes.

The reverse diffusion process is parameterized by an image-domain denoising network ${\epsilon_\theta}$, which predicts the noise component in the noisy volume conditioned on the GARM reconstruction. Starting from Gaussian noise, the model iteratively removes the noise while being guided by the intermediate reconstruction ${I^{\mathrm{rec}}}$. The reverse update at timestep t can be expressed as:

\begin{equation*}{I_{t-1}^{\mathrm{syn}}} = \frac 1{\sqrt{{\alpha_t}}}\left( {I_t^{\mathrm{syn}}}-\frac{1-{\alpha_t}}{\sqrt{1-{\bar{\alpha}_t}}} { \epsilon_\theta}\left( {I_t^{\mathrm{syn}}},t,{I^{\mathrm{rec}}} \right) \right)+{\sigma_t}z,z \sim \mathrm{N}\left( 0,\mathbf{I} \right),\end{equation*}

where ${\sigma_t}$ denotes the variance term of the reverse diffusion step. The condition ${I^{\mathrm{rec}}}$ is critical here because it provides a geometry-informed anatomical initialization rather than a purely data-driven prior. Since ${I^{\mathrm{rec}}}$ is reconstructed from the synthesized full-view projections through the GARM, it already encodes the scanner geometry and coarse anatomical layout implied by the projection measurements. The VRM therefore learns not arbitrary image generation, but conditional volumetric refinement under a physically grounded initialization. This significantly reduces the ambiguity of direct limited-angle image synthesis and enables the model to focus on correcting residual streaking, shading, and local structural degradation that remain after the analytic reconstruction step.

The VRM is trained by minimizing the discrepancy between the sampled Gaussian noise and the noise estimated by the denoising network. Its optimization objective is written as:

\begin{equation*}{L_{\mathrm{VRM}}} = {\mathrm{E}_{{\mathrm{I}_0^{\mathrm{real}}},{\mathrm{I}^{\mathrm{rec}}},t, \epsilon }}\left[\| \epsilon -{ \epsilon_\theta}{\left( {I_t^{\mathrm{real}}},t,{I^{\mathrm{rec}}} \right)\|_2^2} \right].\end{equation*}

During inference, ${P_T^{\mathrm{syn}}}$ and ${I_T^{\mathrm{syn}}}$ are initialized from Gaussian noise and are iteratively denoised by the reverse processes in equations (3) and (7), respectively. Thus, the superscript ‘real’ identifies the clean/noised training targets, whereas ‘syn’ identifies variables generated along the reverse trajectory.

Under this objective, the model learns the reverse denoising trajectory from noisy image samples toward clean target volumes while remaining anchored to the intermediate reconstruction generated by the GARM. In practice, this enables the VRM to recover fine anatomical structures and reduce residual artifacts more effectively than direct analytic reconstruction alone. The final output ${I_0^{\mathrm{syn}}}$ thus represents the refined CBCT volume produced by the PCGA-Net framework.

In addition to standard conditional denoising, the VRM also participates in the cycle-domain supervision mechanism of PCGA-Net. Specifically, the refined or intermediate image prediction can be forward-projected through the GARM to generate projection-domain feedback, which is then used to guide the PCM in subsequent refinement steps. In the training stage, this interaction is reflected through cycle-supervised variables such as ${I_t^{\mathrm{cycle}}}$ and ${I_0^{\mathrm{cycle}}}$, which enforce compatibility between image-domain synthesis and projection-domain consistency under the same acquisition geometry. In the generation stage, the same cross-domain interaction allows the image and projection branches to iteratively support one another, as illustrated in figure 2. This reciprocal design is important because it prevents the VRM from behaving as an isolated post-processing network; instead, it refines the volume while remaining coupled to the projection evidence through the GARM and cycle-domain feedback.

A key practical consequence of this design is that the VRM and PCM have complementary but non-redundant roles. The PCM addresses incompleteness where it physically originates, namely in projection space, whereas the VRM addresses the residual anatomical and textural errors that persist after geometric reconstruction into image space. By conditioning the image-domain diffusion process on ${I^{\mathrm{rec}}}$ rather than on the raw limited-angle input alone, the framework provides the VRM with a substantially stronger and more structured initialization. This division of labor makes the overall PCGA-Net pipeline easier to interpret and better aligned with the physics of limited-angle CBCT reconstruction, where data incompleteness and image degradation arise in related but distinct domains.

#### 2.2.4. Cross-domain geometric coupling and cycle supervision

A central component of PCGA-Net is that the PCM and VRM are not trained as two isolated modules. Instead, they are coupled through a geometry-guided cross-domain cycle supervision strategy, which uses the GARM to establish a closed projection-to-image-to-projection loop under the same cone-beam acquisition geometry. The goal of this design is to ensure that the synthesized full-view projections generated by the PCM can be reconstructed into anatomically meaningful CBCT volumes, while the refined image-domain outputs produced by the VRM remain physically compatible with the projection measurements. As illustrated in figures 1(a)–(c), this supervision is introduced during training by linking projection synthesis, GARM-based reconstruction, and image refinement into one unified optimization framework. During application, the same cross-domain interaction is maintained in the iterative generation procedure shown in figures 2(c)–(a).

In the geometry-conditioned forward path, the PCM first generates the synthetic full-view projection ${P_0^{\mathrm{syn}}}$ from the limited-angle input ${P_0^{\mathrm{real}}}$. The GARM then reconstructs ${P_0^{\mathrm{syn}}}$ into an intermediate CBCT volume ${I^{\mathrm{rec}}}$, which is used as a strict condition for the VRM. To ensure that this intermediate reconstruction is geometrically meaningful, the GARM reconstruction from synthetic projections is supervised against the corresponding reconstruction from the real full-view projections. Let ${I^{\mathrm{real-rec}}}$ denote the CBCT reconstructed from the ground-truth full-view projection $F{P^{\mathrm{real}}}$ through the same GARM. Using mean absolute error (MAE), the reconstruction supervision is defined as ${L_{{\text{CT - rec}}}} = {\mathrm{MAE}}\left( {{I^{{\text{real - rec}}}},{I^{{\mathrm{rec}}}}} \right)$.

This term constrains the PCM and GARM together, encouraging the synthesized projection ${P_0^{\mathrm{syn}}}$ to preserve the anatomical and geometric information necessary for accurate volumetric reconstruction.

On the image side, the VRM receives ${I^{\mathrm{rec}}}$ as the strict conditioning input and progressively generates the refined CBCT output. Under cycle supervision, the corresponding image-domain variable is denoted as ${I_t^{\mathrm{cycle}}}$, and the final cycle-guided output is denoted as ${I_0^{\mathrm{cycle}}}$. This final image is directly supervised against the ground-truth full-scan CT volume ${I_0^{\mathrm{real}}}$ using a voxel-wise reconstruction term, ${\mathrm{L}_{\mathrm{CT-cycle}}} = \mathrm{M}\mathrm{A}\mathrm{E}\left( {I_0^{\mathrm{real}}},{I_0^{\mathrm{cycle}}} \right)$.

This loss enforces that the entire projection-to-image pathway, starting from limited-angle input and passing through projection completion and GARM reconstruction, ultimately converges to a CBCT volume that remains anatomically consistent with the reference CT.

The overall optimization for the image reconstruction branch combines the VRM diffusion losses with the GARM-based reconstruction and cycle supervision terms. Following the notation of the current framework, the total image-side objective is written as:

\begin{equation*}{L_{\mathrm{CT}}} = {L_\theta^{\mathrm{I}\mathrm{,}\mu}}+{\gamma_1}{\mathrm{L}_\theta^{\mathrm{I}\mathrm{,}\Sigma}}+{\gamma_2}{L_{\mathrm{CT-rec}}}+{\gamma_3}{L_{\mathrm{CT-cycle}}},\end{equation*}

where ${\mathrm{L}_\theta^{\mathrm{I}\mathrm{,}\mu}}$ and ${\mathrm{L}_\theta^{\mathrm{I,}\Sigma}}$ denote the mean and variance prediction losses in the VRM, respectively, and ${\gamma_1}$, ${\gamma_2}$, and ${\gamma_3}$ (0.05, 0.5 and 0.5) are empirical weighting coefficients. In this formulation, the diffusion model is not trained only to denoise image-domain samples, but also to remain compatible with the GARM-reconstructed intermediate volume and the underlying projection geometry.

The coefficients were selected empirically in preliminary training and validation runs to keep the contributions of the diffusion and geometry-based objectives on comparable numerical scales. Specifically, ${\gamma_1} = 0.05$ downweights the variance/variational lower bound term relative to the principal mean-prediction objective, whereas ${\gamma_2} = {\gamma_3} = 0.5$ assign comparable influence to the GARM reconstruction and cycle-consistency terms. These values were fixed before test-set evaluation and were not tuned using test performance.

The importance of this cross-domain supervision is that it prevents the full framework from degenerating into a loosely connected cascade. Without ${\mathrm{L}_{\mathrm{CT-rec}}}$, the PCM could generate projection completions that appear plausible in projection space but are suboptimal for downstream volumetric reconstruction. Without ${\mathrm{L}_{\mathrm{CT-cycle}}}$, the VRM could refine ${I^{\mathrm{rec}}}$ in a way that improves visual appearance while drifting away from the anatomical structure implied by the projection data. By coupling projection synthesis, GARM reconstruction, and image refinement through the same geometry-guided cycle, PCGA-Net enforces that both domains converge toward a mutually compatible solution. This design is particularly important in limited-angle CBCT, where the missing information originates in projection space, but its clinical consequence appears in image space as shading, streaking, and structural distortion.

#### 2.2.5. Inference process

Figures 1 and 2 describe different stages of PCGA-Net. Figure 1 summarizes supervised training and therefore includes clean ground-truth targets, forward-diffusion training states, and optimization losses. Figure 2 contains none of these training-only quantities; instead, it unrolls one application-time transition from the current reverse timestep s to the subsequent timestep s-1, showing how deterministic estimates provide geometry-mediated cross-domain feedback while stochastic states advance the reverse diffusion trajectories. The trained model is therefore applied iteratively rather than as a single-pass PCM–GARM–VRM cascade.

After training, PCGA-Net reconstructs limited-angle CBCT through an iterative application process that alternates between the PCM, the GARM, and the VRM. Unlike a one-pass feedforward pipeline, the proposed application strategy preserves the same cross-domain geometric linkage used during training, allowing the projection and image branches to progressively support one another during reverse diffusion. As illustrated in figures 2(c)–(a), the generation starts from noisy projection and image variables and then proceeds through reciprocal refinement in projection space and image space under GARM guidance. This design is particularly important for limited-angle CBCT, because the missing-angle information recovered in projection space and the anatomical structures refined in image space must remain compatible throughout the sampling trajectory rather than only at the final output.

At the beginning of application, the PCM and VRM are initialized with noisy variables at the starting timestep s=S, denoted as ${P_S}$ and ${I_S}$, respectively. During the early reverse steps, both branches operate mainly under weak conditions to improve efficiency, since the synthesized projections and images at high noise levels contain limited anatomical information. In this stage, the noisy projection ${P_s}$ is denoised by the PCM conditioned on the measured limited-angle input ${P_0^{\mathrm{real}}}$, and the noisy image ${I_s}$ is denoised by the VRM conditioned on the GARM-based initialization. Following the notation of the current framework, the stochastic reverse sampling can be expressed generically as:

\begin{equation*}{X_{s-1}^{\mathrm{sto}}} = {\mu_s^\theta}\left( {X_s},s,Z \right)+{\Sigma_s^\theta}\left( {X_s},s,Z \right) {z_s},{z_s} \sim \mathrm{N}\left( 0,I \right),\end{equation*}

where ${X_s}$ denotes either the projection variable or the image variable, and Z denotes the corresponding conditioning input. In addition to stochastic sampling, each DDPM also produces a deterministic output at the same timestep, which is used to establish the geometry-guided interaction between the two branches.

Once the reverse process reaches the geometry-guided stage, the PCM first generates the deterministic full-view projection ${P_{s-1}^{\mathrm{det}}}$ from the current noisy state. This synthesized projection is then reconstructed through the GARM to obtain the intermediate CBCT volume ${I_{s-1}^{\mathrm{rec}}}$. As shown in figures 2(c) and (b), ${I_{s-1}^{\mathrm{rec}}}$ serves as the strict condition for the VRM, allowing the image branch to refine its current noisy estimate and generate both a deterministic image ${I_{s-1}^{\mathrm{det}}}$ and a stochastic image ${I_{s-1}^{\mathrm{sto}}}$. The deterministic image output is further forward-projected through the GARM to produce ${P_{s-1}^{\mathrm{rec}}}$, which is then used as a stricter geometric reference for the PCM at the subsequent timestep. In this way, the application process forms a repeated loop, thereby establishing the projection-to-image-to-projection interaction throughout reverse generation.

More specifically, the PCM at timestep s uses both the measured limited-angle input ${P_0^{\mathrm{real}}}$ and the GARM-derived reference ${P_s^{\mathrm{rec}}}$ to generate the projection variables for timestep s−1. The resulting deterministic output ${P_{s-1}^{\mathrm{det}}}$ provides the geometrically completed projection used for reconstruction, while the stochastic output ${P_{s-1}^{\mathrm{sto}}}$ is propagated to the next reverse step. Similarly, the VRM uses the GARM reconstruction ${I_{s-1}^{\mathrm{rec}}}$ as the strict condition to generate ${I_{s-1}^{\mathrm{det}}}$ and ${I_{s-1}^{\mathrm{sto}}}$. This alternating deterministic-stochastic design is consistent with the application scheme in our prior geometry-integrated diffusion frameworks, but here it is adapted to continuous limited-angle acquisition, where the local angular continuity of the short-arc data and the global incompleteness of the unscanned sector must be handled simultaneously.

By recursively applying this geometric feedback loop from timestep S to timestep 0, the framework progressively improves both the synthesized projection and the reconstructed image. The final output of the application process is the clean CBCT volume ${I_0^{\mathrm{syn}}}$, which is obtained after the VRM completes the last reverse step under the strict condition derived from the GARM. Because the projection branch and image branch are refined jointly rather than independently, the final CBCT output benefits from both projection-domain missing-angle compensation and image-domain anatomical restoration. This application strategy is therefore a key part of PCGA-Net: it extends the geometry-guided coupling introduced during training into the reverse generation stage, ensuring that the final reconstruction remains physically grounded, anatomically coherent, and consistent with the measured limited-angle projections.

## 3. Experimental setup

### 3.1. Datasets, split, and preprocessing

We evaluated PCGA-Net in three clinically distinct cohorts with different anatomical complexity, motion characteristics, and image-guidance workflows: GYN HDR brachytherapy pelvic CT, abdominal liver 4DCT, and H&N CBCT. This multi-site design was intended to assess whether the proposed framework remains effective across procedure-oriented pelvic imaging, respiration-affected upper-abdominal imaging, and anatomically intricate on-board H&N imaging, rather than in a single disease site alone. Consistent with the overall goal of balancing cross-patient generalizability and patient-specific reconstruction fidelity, we adopted a two-stage training strategy for all three cohorts: a population-level pretraining stage to learn anatomy- and geometry-related priors shared across patients, followed by a patient-specific few-shot adaptation stage to tailor the model to the target patient. In our design a global prior is first learned from a broader cohort and then refined for patient-specific reconstruction.

For the GYN HDR brachytherapy cohort, data from 30 patients were used for population-level pretraining, and an additional 30 patients were reserved for adaptation and evaluation. For each patient in the adaptation cohort, 2 early fractions were used for patient-specific fine-tuning, and the remaining 3 fractions were used for testing. This cohort represents a challenging pelvic reconstruction scenario in which volumetric imaging is closely associated with applicator or needle verification, target localization, and peri-procedural assessment. Each fraction contained 229 slices with an in-plane matrix size of 512 × 512. The original scans were acquired on a Siemens Healthineers SOMATOM go. Open Pro CT system (120 kVp, 126 mA, 157 mAs), with 1.0 mm slice thickness and pixel spacing of 0.3774 × 0.3774 mm^2^. The corresponding geometric parameters used to define the simulated cone-beam configuration were SDD = 1113 mm and source-to-axis distance (SAD) = 610 mm. For this cohort, CT volumes were used to generate simulated cone-beam projections under a predefined geometry, enabling controlled evaluation against artifact-free volumetric references.

For the abdominal liver 4DCT cohort, 50 patients were included in the pretraining stage, with 10 respiratory phases per patient used to capture shared respiratory-motion priors. The adaptation cohort consisted of 15 patients, for whom 3 phases/fractions were used for patient-specific fine-tuning and 6 phases/fractions were used for testing. This cohort was selected because limited-angle reconstruction in the upper abdomen is complicated by low soft-tissue contrast and respiration-driven deformation, making accurate recovery of volumetric anatomy particularly demanding. Each volume contained 178 slices with a 512 × 512 in-plane matrix. The 4DCT scans were acquired on a Siemens SOMATOM Definition Edge scanner (120 kVp) with 2.0 mm slice thickness and pixel spacing of 0.9766 × 0.9766 mm^2^. The corresponding geometric parameters were SDD = 1085.6 mm and SAD = 595 mm. As in the GYN cohort, the 4DCT volumes were used as artifact-reduced volumetric references from which cone-beam projections were simulated. This strategy is advantageous for CBCT reconstruction research because it provides exactly paired projection-volume data under known acquisition geometry, which is difficult to guarantee using real CBCT alone.

For the H&N cohort, 40 patients were used for pretraining and 16 patients for patient-specific adaptation and testing. Because this cohort contains the most complex osseous-air-soft-tissue interfaces and is strongly influenced by immobilization-related geometry, a deeper adaptation setting was used: 10 fractions per patient were used for fine-tuning and the remaining 5 fractions were used for evaluation. This cohort represents a realistic daily on-board image-guided radiotherapy setting, where CBCT is clinically used for setup verification, monitoring of treatment-induced anatomical changes, and adaptive decision-making. Each fraction contained 106 slices with a 512 × 512 in-plane matrix. The CBCT scans were acquired on a Varian Medical Systems P2VA system (100 kVp, 154 mA, 889 mAs), with slice thickness of 1.9964 mm, pixel spacing of 0.5655 × 0.5655 mm^2^, SDD = 3700 mm, and SAD = 2700 mm. Unlike the GYN and abdominal cohorts, this dataset was evaluated directly in the CBCT domain because matched planning CT volumes were not consistently available for all cases. This complementary design allowed us to test PCGA-Net in both CT-based projection simulation settings and real clinical CBCT acquisitions.

All projection–domain inputs in this study were generated under a prescribed cone-beam geometry. For the GYN and liver cohorts, CT or 4DCT volumes were forward-projected to generate paired full-view and limited-angle data. For the H&N cohort, reconstructed clinical on-board CBCT volumes served as the source volumes and reference targets. Because vendor raw detector projections were not available, each reconstructed H&N CBCT volume was forward-projected with LEAP-CT to generate a synthetic 360-view projection stack, from which the fixed 90-view short arc was retained as the model input. Therefore, the H&N experiment uses clinically acquired reconstructed CBCT anatomy but simulated projection measurements; it is not an evaluation on original raw detector data.

All datasets underwent consistent preprocessing prior to model training. First, both projection data and volumetric images were intensity-normalized to [−1, 1], to stabilize optimization and match the operating range of the diffusion model. During inference, the reconstructed outputs were mapped back to the original Hounsfield unit (HU) scale for quantitative evaluation. Second, all images were resampled and cropped to a unified in-plane matrix size of 512 × 512. The slice direction was downsampled or standardized to fit graphics processing unit (GPU) memory while preserving the major anatomical structures, resulting in an anisotropic resolution close to 1 × 1 × 2 mm^3^. Third, a body mask was applied to remove background air and restrict analysis to the patient body region, thereby reducing irrelevant background variation during training and evaluation. For the CT-based cohorts, patient-specific cone-beam geometry was constructed from the corresponding Digital Imaging and Communications in Medicine (DICOM) metadata, and full-view projections were simulated using the same geometric configuration employed by the GARM. Limited-angle inputs were then generated by retaining only the designated short-arc views for model input, while the full-view projections were used as supervision targets.

The full-view projection stack contained 360 uniformly spaced views, with angular positions ${\theta_k} = {k^\circ}$ for $k = 0, \ldots ,{\mathrm{359}}$. The limited-angle input retained 90 consecutive clockwise views defined by ${\theta_k} = {\left( 135+k \right)^\circ}$ for $k = 0, \ldots ,{\mathrm{89}}$. Thus, the discrete short-arc set covers the nominal half-open interval $[13{5^\circ},22{5^\circ})$ at ${1^\circ}$ spacing. This identical angular subset was used to generate the limited-angle inputs for PCGA-Net, FDK, cGAN, and PatRecon-Net.

The simulated projection stacks were generated as deterministic forward projections. No additional Poisson photon noise or Gaussian electronic noise was injected after projection generation. This design was selected to isolate reconstruction errors caused by angular incompleteness from those caused by stochastic measurement noise. The same assumption was used for the CT/4DCT-derived projections and for the re-projected H&N CBCT volumes.

The multi-site evaluation assesses cross-site robustness under the specified two-stage protocol rather than patient-independent zero-shot generalization. For each anatomical site, a population-level model was first trained using a separate pretraining cohort. For each target patient, only designated early fractions or respiratory phases were then used for patient-specific adaptation, and the remaining later fractions or phases were held out for evaluation. The GYN, liver, and H&N adaptation sets contained 2, 3, and 10 early fractions/phases per patient, respectively; the corresponding held-out test sets contained 3, 6, and 5 later fractions/phases. No projection, volume, or supervision target derived from a held-out test fraction/phase was used during adaptation.

### 3.2. Implementation details

All model training and inference were conducted on a workstation equipped with two NVIDIA RTX PRO 6000 Blackwell Max-Q GPUs, providing a total of 192 GB GDDR7 memory. This hardware configuration enabled efficient training of the dual-domain diffusion framework with mixed-precision computation, including bfloat16 (BF16) and 8-bit floating-point (FP8) precision. The software stack was built on PyTorch 2.4, and the GARM was implemented using LEAP-CT to provide differentiable forward- and backward-projection operators under the predefined cone-beam geometry. This configuration was selected to support memory-intensive 3D diffusion training while preserving end-to-end geometric consistency across the projection and image domains.

Training was performed on randomly sampled three-dimensional subvolumes of $64\times 64\times 16$ voxels. Accordingly, the reported batch size of 16 denotes the effective number of subvolumes per optimization step rather than 16 complete 512×512 volumes. Memory use was further controlled through mixed-precision computation and alternating PCM/VRM updates, during which gradients were retained only for the branch being optimized. During evaluation, the standardized volume was processed using sliding-window inference with normalized Gaussian weighting, avoiding manual hard concatenation of independently reconstructed blocks.

Training was performed in two stages: foundation pretraining and patient-specific fine-tuning. In the first stage, a population-level reconstruction model was pretrained separately for each anatomical site using the corresponding pretraining cohort, namely 30 patients for GYN HDR brachytherapy, 50 patients for liver 4DCT, and 40 patients for H&N CBCT. The purpose of this stage was to learn anatomy-aware image priors and short-arc projection completion patterns shared across patients within each site. Model optimization was performed using the AdamW optimizer with an initial learning rate of $2\times 1{0^{-5}}$, and the total diffusion length was set to T=1000 with a linear noise schedule. Benefiting from the large GPU memory available on the Blackwell platform, the batch size was set to 16, and pretraining was continued for up to 500 epochs or until the validation loss reached convergence. We employed an early stopping strategy with a patience of 20 epochs and a minimum improvement threshold of 0.001. This stage produced the initialization weights for the subsequent patient-specific adaptation step.

In the second stage, the pretrained model weights were loaded and fully fine-tuned for each patient in the adaptation cohort using only a small number of early fractions or phases. This patient-specific full-parameter adaptation was introduced to reduce residual anatomical mismatch between the population model and the target patient, and to better accommodate patient-dependent imaging appearance, motion pattern, and scanner-specific noise characteristics. During fine-tuning, the learning rate was reduced to $1\times 1{0^{-5}}$ to ensure stable adaptation, and the number of training epochs was set to 100. All model parameters were updated during this stage rather than freezing selected layers, because the projection and image diffusion branches, as well as their GARM-mediated interaction, all contribute to patient-specific reconstruction fidelity. This design follows the same overall principle as in our prior geometry-integrated CBCT reconstruction studies, where a global model first captures shared anatomical structure and is then adapted to the target patient using a small number of available images.

During the 100-epoch patient-specific adaptation stage, supervised pairs were constructed only from the designated early fractions/phases. For the PCM, the 90-view limited-angle projection served as the conditional input and the paired full-view projection stack served as the target. For the VRM, the GARM-derived intermediate reconstruction served as the conditional input and the corresponding reference CT/4DCT/CBCT volume served as the image target; the same early-fraction references were used for the GARM reconstruction and cycle-consistency terms. The later fractions/phases reserved for testing were not used for parameter optimization. Accordingly, the reported results represent a longitudinal patient-adapted operating point in which prior images from the same patient are available.

The overall training objective combined the diffusion losses in projection and image space with the GARM-based reconstruction and cycle-supervision terms. During implementation, no weight decay and gradient clipping were applied as needed to stabilize diffusion training on volumetric data. Loss updates alternated between the projection and image branches, assigning zero gradients to the uninvolved network during each update step. At inference time, the same pretrained-and-adapted model was used to reconstruct limited-angle CBCT through the geometry-guided reverse generation process, with 50 reverse sampling steps (using a timestep respacing strategy) to balance reconstruction accuracy and computational efficiency.

The PCM and VRM are implemented as distinct three-dimensional Swin-Vnet conditional denoisers. Each denoiser follows a seven-stage encoder and seven-stage decoder design with resolution-matched skip connections and three Swin-attention blocks at the bottleneck. Instance normalization and ReLU activation are applied after the convolutional and attention layers. Diffusion time is represented by a 128-dimensional sinusoidal embedding with a maximum period of 1024 and is injected into the network blocks. The decoder output heads predict the parameters required for the mean and variance terms of reverse diffusion. The PCM receives the noisy full-view projection state together with the measured 90-view short-arc projection stack and synthesizes the completed projection representation used by the GARM. The VRM receives the noisy image-domain state together with the GARM-derived intermediate reconstruction and refines the volume toward the target CBCT distribution. Complete functional definitions of the PCM, GARM, and VRM, together with channel widths, convolution kernels, anisotropic down- and up-sampling factors, cumulative relative resolutions, bottleneck attention settings, and memory-handling details, are provided in supplementary tables S1–S4.

For the cGAN comparison, we used a volumetric pix2pix-style image-domain implementation. The input was the FDK volume reconstructed from the same 90-view short arc used by PCGA-Net, and the supervision target was the corresponding full-view/reference CBCT. The generator followed a three-dimensional U-Net-like encoder-decoder and was trained with a three-dimensional PatchGAN discriminator using adversarial and voxel-wise ${L_1}$ reconstruction objectives. The same cohort partitions, preprocessing, body mask, and evaluation protocol were used for all methods. For the fully adapted comparisons in tables 2–4, cGAN, PatRecon-Net, and PCGA-Net each underwent patient-specific fine-tuning using the identical designated early fractions/phases and the same fine-tuning settings as PCGA-Net. Each learned model was initialized from its corresponding site-specific population checkpoint and adapted for 100 epochs with a learning rate of $1\times 1{0^{-5}}$ and full-parameter updates. The cohort-specific adaptation sets comprised 2 prior fractions for GYN, 10 for H&N, and 3 fractions/phases for liver. All methods were evaluated on the same held-out later fractions/phases. Table 5 separately presents the population-only (N=0) and fully adapted operating points; N=0 denotes use of the population checkpoint before target-patient fine-tuning.

**Table 2.** Quantitative comparison of limited-angle CBCT reconstruction performance on the GYN HDR brachytherapy CT cohort. All learned methods (cGAN, PatRecon-Net, and PCGA-Net) were fine-tuned on the same 2 prior fractions from each target patient and evaluated on the same 3 held-out test fractions. The fine-tuning settings matched those of PCGA-Net: 100 epochs, a learning rate of $1\times 1{0^{-5}}$, and full-parameter updates. FDK is an analytic baseline without fine-tuning. Results are reported as mean ± standard deviation over the test fractions. MAE is expressed in Hounsfield units (HU), PSNR in decibels (dB), and SSIM as a unitless structural similarity measure. The symbols ↓ and ↑ indicate that lower and higher values are preferred, respectively. * indicates statistical significance with p<0.05 compared with the proposed PCGA-Net. Bold values indicate the best performance.

Method	MAE (HU) ↓	SSIM ↑	PSNR (dB) ↑
FDK (Baseline)	178.63 ± 31.58*	0.51 ± 0.03*	18.97 ± 1.31*
cGAN	62.45 ± 10.12*	0.71 ± 0.05*	29.30 ± 1.55*
PatRecon-Net	48.90 ± 8.76*	0.78 ± 0.03*	31.85 ± 1.30*
**PCGA-Net (Ours)**	**34.47 ± 6.28**	**0.87 ± 0.04**	**35.79 ± 0.95**

**Table 3.** Quantitative comparison of limited-angle CBCT reconstruction performance on the H&N CBCT cohort. All learned methods (cGAN, PatRecon-Net, and PCGA-Net) were fine-tuned on the same 10 prior fractions from each target patient and evaluated on the same 5 held-out test fractions. The fine-tuning settings matched those of PCGA-Net: 100 epochs, a learning rate of $1\times 1{0^{-5}}$, and full-parameter updates. FDK is an analytic baseline without fine-tuning. Results are reported as mean ± standard deviation over the test fractions. MAE is expressed in Hounsfield units (HU), PSNR in decibels (dB), and SSIM as a unitless structural similarity measure. The symbols ↓ and ↑ indicate that lower and higher values are preferred, respectively. * indicates statistical significance with p<0.05 compared with the proposed PCGA-Net. Bold values indicate the best performance.

Method	MAE (HU) ↓	SSIM ↑	PSNR (dB) ↑
FDK (Baseline)	165.30 ± 25.40*	0.61 ± 0.05*	21.13 ± 1.14*
cGAN	55.18 ± 8.25*	0.79 ± 0.04*	31.49 ± 1.43*
PatRecon-Net	41.87 ± 7.51*	0.85 ± 0.03*	34.10 ± 1.25*
**PCGA-Net (Ours)**	**28.42 ± 4.88**	**0.91 ± 0.05**	**38.52 ± 0.95**

**Table 4.** Quantitative comparison of limited-angle CBCT reconstruction performance on the abdominal liver 4DCT cohort. All learned methods (cGAN, PatRecon-Net, and PCGA-Net) were fine-tuned on the same 3 prior fractions/phases from each target patient and evaluated on the same 6 held-out test fractions/phases. The fine-tuning settings matched those of PCGA-Net: 100 epochs, a learning rate of $1\times 1{0^{-5}}$, and full-parameter updates. FDK is an analytic baseline without fine-tuning. Results are reported as mean ± standard deviation over the test fractions/phases. MAE is expressed in Hounsfield units (HU), PSNR in decibels (dB), and SSIM as a unitless structural similarity measure. The symbols ↓ and ↑ indicate that lower and higher values are preferred, respectively. * indicates statistical significance with p<0.05 compared with the proposed PCGA-Net. Bold values indicate the best performance.

Method	MAE (HU) ↓	SSIM ↑	PSNR (dB) ↑
FDK (Baseline)	195.21 ± 40.08*	0.50 ± 0.06*	18.50 ± 1.50*
cGAN	68.14 ± 12.32*	0.68 ± 0.07*	28.15 ± 1.87*
PatRecon-Net	53.42 ± 9.51*	0.75 ± 0.05*	30.90 ± 1.45*
**PCGA-Net (Ours)**	**39.27 ± 6.10**	**0.82 ± 0.03**	**36.85 ± 1.16**

**Table 5.** Population-only and fully adapted reconstruction performance across the three cohorts. N denotes the number of target-patient prior fractions/phases used for adaptation. Population-only inference (N=0) uses the site-specific population-pretrained model without target-patient fine-tuning. Fully adapted learned models use the same designated prior fractions/phases and matched adaptation budgets within each cohort, with identical held-out test fractions/phases. Values are cohort-level means; fully adapted means correspond to tables 2–4. For MAE and SSIM, parentheses give the relative percentage change from the same model’s population-only result, calculated as $100\left( {m_{\mathrm{adapted}}}-{m_{N = 0}} \right)/{m_{N = 0}}$. For PSNR, parentheses give the absolute difference in dB, calculated as the fully adapted value minus the population-only value. MAE is reported in HU and PSNR in dB; SSIM is unitless. Negative MAE changes and positive SSIM/PSNR changes indicate improvement. FDK has no adaptation stage; its reference results are provided in tables 2–4.

		Population-only (N=0)			Fully adapted		
Cohort	Method	MAE ↓	SSIM ↑	PSNR ↑	MAE ↓	SSIM ↑	PSNR ↑
GYN HDR brachytherapy ${N_{\mathrm{full}}} = 2$	cGAN	85.0	0.65	26.5	62.45 (−26.53%)	0.71 (+9.23%)	29.30 (+2.80 dB)
	PatRecon-Net	70.0	0.74	30.5	48.90 (−30.14%)	0.78 (+5.41%)	31.85 (+1.35 dB)
	PCGA-Net	58.06	0.79	33.29	34.47 (−40.63%)	0.87 (+10.13%)	35.79 (+2.50 dB)

Head-and-neck CBCT ${N_{\mathrm{full}}} = 10$	cGAN	76.0	0.70	28.0	55.18 (−27.39%)	0.79 (+12.86%)	31.49 (+3.49 dB)
	PatRecon-Net	62.0	0.76	31.0	41.87 (−32.47%)	0.85 (+11.84%)	34.10 (+3.10 dB)
	PCGA-Net	47.88	0.80	33.37	28.42 (−40.64%)	0.91 (+13.75%)	38.52 (+5.15 dB)

Abdominal liver 4DCT ${N_{\mathrm{full}}} = 3$	cGAN	112.0	0.60	25.0	68.14 (−39.16%)	0.68 (+13.33%)	28.15 (+3.15 dB)
	PatRecon-Net	96.0	0.66	28.5	53.42 (−44.35%)	0.75 (+13.64%)	30.90 (+2.40 dB)
	PCGA-Net	82.66	0.71	30.75	39.27 (−52.49%)	0.82 (+15.49%)	36.85 (+6.10 dB)

PatRecon-Net was implemented using the authors’ public repository. Its data loader accepts a user-defined number of views, stacks the projections along the input-channel dimension, and constructs ReconNet with the input-channel count set to num_views. We therefore set num_views = 90 and supplied the measured short-arc projections in acquisition order, while retaining the reference 3D volume as the reconstruction target. No projection-completion branch, analytic GARM, or cycle-domain coupling was added.

### 3.3. Evaluation metrics

Reconstruction performance was assessed using three standard image-quality metrics: MAE, peak signal-to-noise ratio (PSNR), and structural similarity index measure (SSIM). These metrics were selected because they provide complementary information on voxel-wise intensity fidelity, global signal reconstruction quality, and structural preservation, respectively, and are widely used for quantitative evaluation in CBCT reconstruction. In all experiments, the reconstructed limited-angle CBCT volume was compared against the corresponding reference volume on a voxel-by-voxel basis after restoration to the original HU scale. Lower MAE and higher PSNR/SSIM indicate better reconstruction performance. Consistent with the comparative setting of this study, PCGA-Net was quantitatively evaluated against cGAN (Isola *et al* 2017) and PatRecon-Net (Shen *et al* 2019) as learning-based baseline methods, and FDK was used as a physics-based reference.

The MAE was used to measure the average absolute voxel-wise difference between the reconstructed volume ${I^{\mathrm{pred}}}$ and the reference volume ${I^{\mathrm{ref}}}$, and was computed as $\mathrm{MAE=}\frac 1{N}\sum \nolimits_{i = 1}^{N}\left| {I_i^{\mathrm{pred}}}-{I_i^{\mathrm{ref}}} \right|$, where N denotes the total number of voxels within the evaluated body region. MAE directly reflects HU reconstruction accuracy and is therefore particularly relevant for assessing whether the reconstructed CBCT preserves anatomically meaningful attenuation values. The PSNR was used to evaluate the overall signal fidelity of the reconstructed image and was calculated as $\mathrm{PSNR=}10\mathrm{lo}{\mathrm{g}_{10}}\left( \frac{\mathrm{MA}{\mathrm{X}^2}}{\mathrm{MSE}} \right)$, where the mean squared error (MSE) is $\mathrm{MSE=}\frac 1{N}\sum \nolimits_{i = 1}^{N}{\left( {I_i^{\mathrm{pred}}}-{I_i^{\mathrm{ref}}} \right)^2}$, and $\mathrm{MAX}$ denotes the maximum possible intensity value of the reference image. Higher PSNR indicates lower reconstruction error and better overall image fidelity.

To further assess preservation of anatomical structure, SSIM was computed between the reconstructed and reference volumes. SSIM measures local similarity in luminance, contrast, and structural content, and is defined as $\mathrm{SSIM}\left( x,y \right) = \frac{\left( 2{\mu_x}{\mu_y}+{C_1} \right)\left( 2{\sigma_{xy}}+{C_2} \right)}{\left( {\mu_x^2}+{\mu_y^2}+{C_1} \right)\left( {\sigma_x^2}+{\sigma_y^2}+{C_2} \right)}$, where ${\mu_x}$ and ${\mu_y}$ are the local means of x and y, ${\sigma_x^2}$ and ${\sigma_y^2}$ are the corresponding local variances, ${\sigma_{xy}}$ is the local covariance, and ${C_1}$ and ${C_2}$ are small constants for numerical stability. In this study, SSIM was calculated over the reconstructed body region to quantify how well the structural organization of the target anatomy was preserved after limited-angle reconstruction. Compared with MAE and PSNR, SSIM is more sensitive to perceptually important structural distortion, making it particularly useful for evaluating artifact suppression and anatomical integrity.

## 4. Results

Across all three datasets, the qualitative comparisons consistently indicate that limited-angle FDK reconstruction remains strongly affected by streaking, shading, and view-dependent structural distortion, although the visual manifestation of these artifacts varies with anatomy, motion pattern, and workflow-specific imaging conditions. In the GYN HDR brachytherapy cohort, the main difficulty lies in recovering applicator-adjacent pelvic soft tissue under crowded procedural anatomy; in the H&N cohort, the challenge is dominated by complex bone-air-soft-tissue interfaces and elongated anatomical structure; and in the abdominal liver 4DCT cohort, limited-angle degradation is compounded by low soft-tissue contrast and respiration-associated anatomical variation. Despite these distinct failure modes, PCGA-Net shows a consistent qualitative advantage over FDK and the compared learning-based baselines by producing anatomically more coherent reconstructions with reduced residual artifacts across axial, coronal, and sagittal views. This cross-dataset visual consistency suggests that the proposed geometry-integrated dual-domain design is not confined to one specific anatomical site, but is capable of handling diverse limited-angle reconstruction scenarios with different structural complexity and clinical imaging characteristics.

### 4.1. Qualitative results

#### 4.1.1. Qualitative assessment of the GYN HDR brachytherapy CT

Representative qualitative results for the GYN HDR brachytherapy cohort are shown in figure 3, including axial, coronal, and sagittal views together with the corresponding error maps. The FDK reconstruction exhibits typical limited-angle degradation across all three planes, with pronounced streaking, shading, and loss of anatomical continuity, particularly around the pelvic midline and applicator-adjacent soft tissues. These artifacts are most evident in the coronal and sagittal views, where depth-direction distortion and structural elongation are clearly visible.

**Figure 3.**
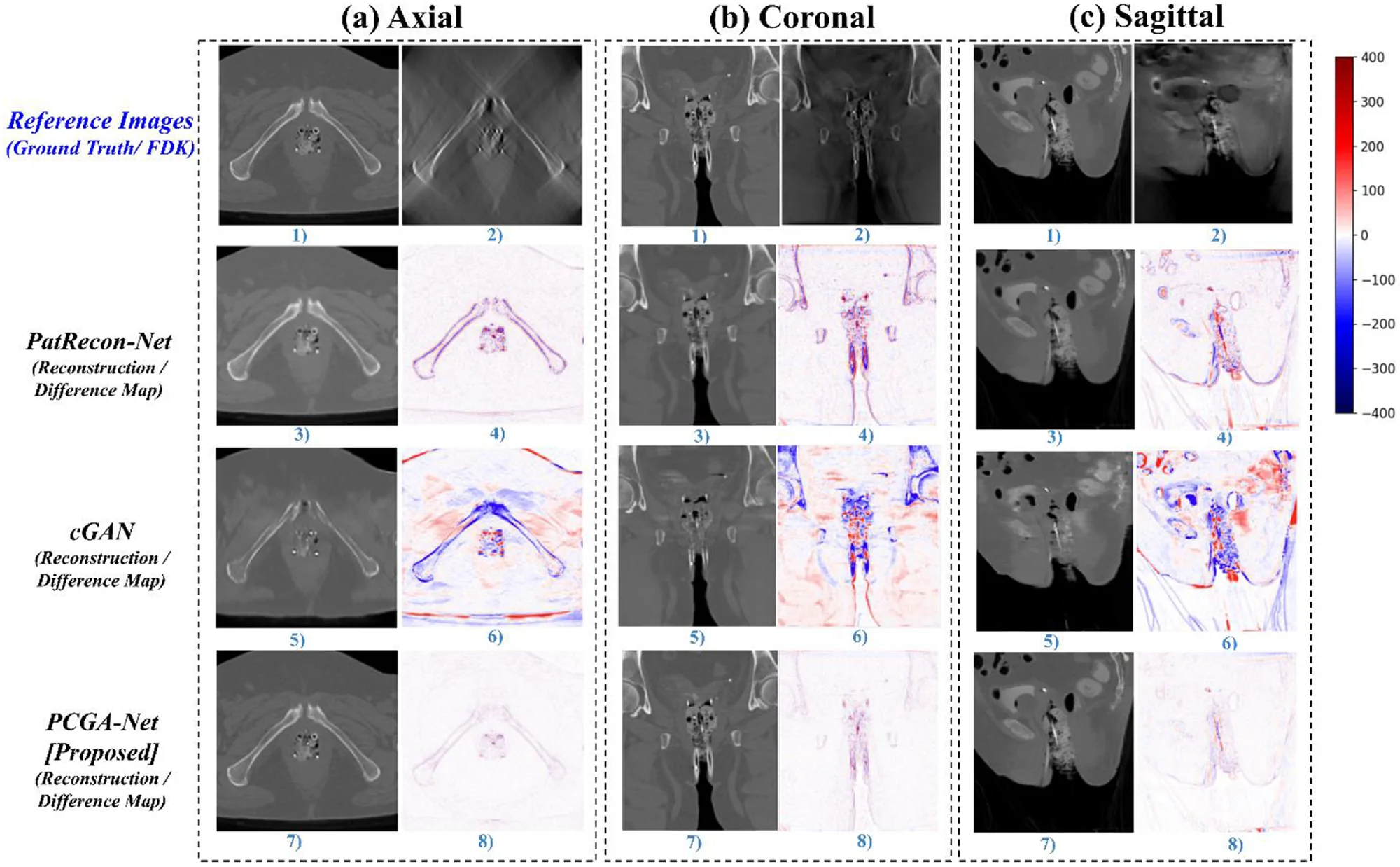
Qualitative comparison of limited-angle CBCT reconstruction methods on a representative gynecologic HDR brachytherapy case. Columns (a)–(c) show the axial, coronal, and sagittal views, respectively. In each column, the first row provides the reference images, where subpanels 1 show the ground-truth CBCT and subpanels 2 show the corresponding 90° limited-angle FDK reconstructions. The remaining rows correspond to PatRecon-Net, cGAN, and the proposed PCGA-Net, respectively; for these methods, subpanels 3, 5, and 7 show the reconstructed images, and subpanels 4, 6, and 8 show the corresponding difference maps with respect to the ground-truth CBCT. Difference maps are displayed using the color scale on the right, with intensity differences ranging from −400 to 400 HU. Compared with the 90° limited-angle FDK reconstruction and the learning-based baselines, PCGA-Net shows reduced residual artifacts and closer anatomical agreement with the ground-truth CBCT across all three anatomical planes.

Among the learning-based methods, PatRecon-Net reduces part of the large-scale artifact pattern relative to FDK, but residual errors remain visible around the pelvic bones and central soft-tissue structures. In contrast, cGAN introduces more substantial intensity deviation and structural inconsistency, with broader high-magnitude error patterns distributed across the body contour and central pelvic region. Compared with both baselines, PCGA-Net provides the closest visual agreement to the reference CBCT. The reconstructed pelvic anatomy is more spatially coherent, the bony contours are better preserved, and the surrounding soft tissues appear less corrupted by limited-angle streaking. This advantage is consistently reflected in the error maps, where PCGA-Net shows the weakest residual deviations in all three anatomical planes.

In addition to the surrounding pelvic anatomy, PCGA-Net also provides more faithful reconstruction of the brachytherapy applicator itself, which is a critical requirement in the GYN HDR setting. In the FDK reconstruction, the applicator region is degraded by strong limited-angle streaking and local shading, making the device boundaries appear blurred and partially distorted. PatRecon-Net mitigates part of this degradation but still shows residual structural ambiguity around the applicator channel and adjacent high-contrast interfaces, while cGAN introduces more noticeable intensity bias and shape inconsistency in the same region. By comparison, PCGA-Net preserves the applicator geometry with sharper definition and reduced surrounding artifact contamination, particularly in the coronal and sagittal views where limited-angle distortion is typically more severe. The corresponding error maps also show that PCGA-Net yields the smallest residual deviation near the applicator, suggesting improved recovery of both the device structure and its spatial relationship to the adjacent pelvic soft tissue.

#### 4.1.2. Qualitative assessment of the H&N CBCT

Representative qualitative results for the H&N cohort are shown in figure 4, where axial, coronal, and sagittal views are presented together with the corresponding error maps. Compared with the reference CBCT, the FDK reconstruction exhibits substantial limited-angle artifacts across all three planes, including strong shading in the central neck region, blurred soft-tissue boundaries, and loss of structural continuity along the cervical spine and airway-adjacent anatomy. These degradations are particularly evident in the coronal and sagittal views, where the missing-angle effect introduces pronounced elongation and nonuniform intensity distortion over the long superior-inferior extent of the neck.

**Figure 4.**
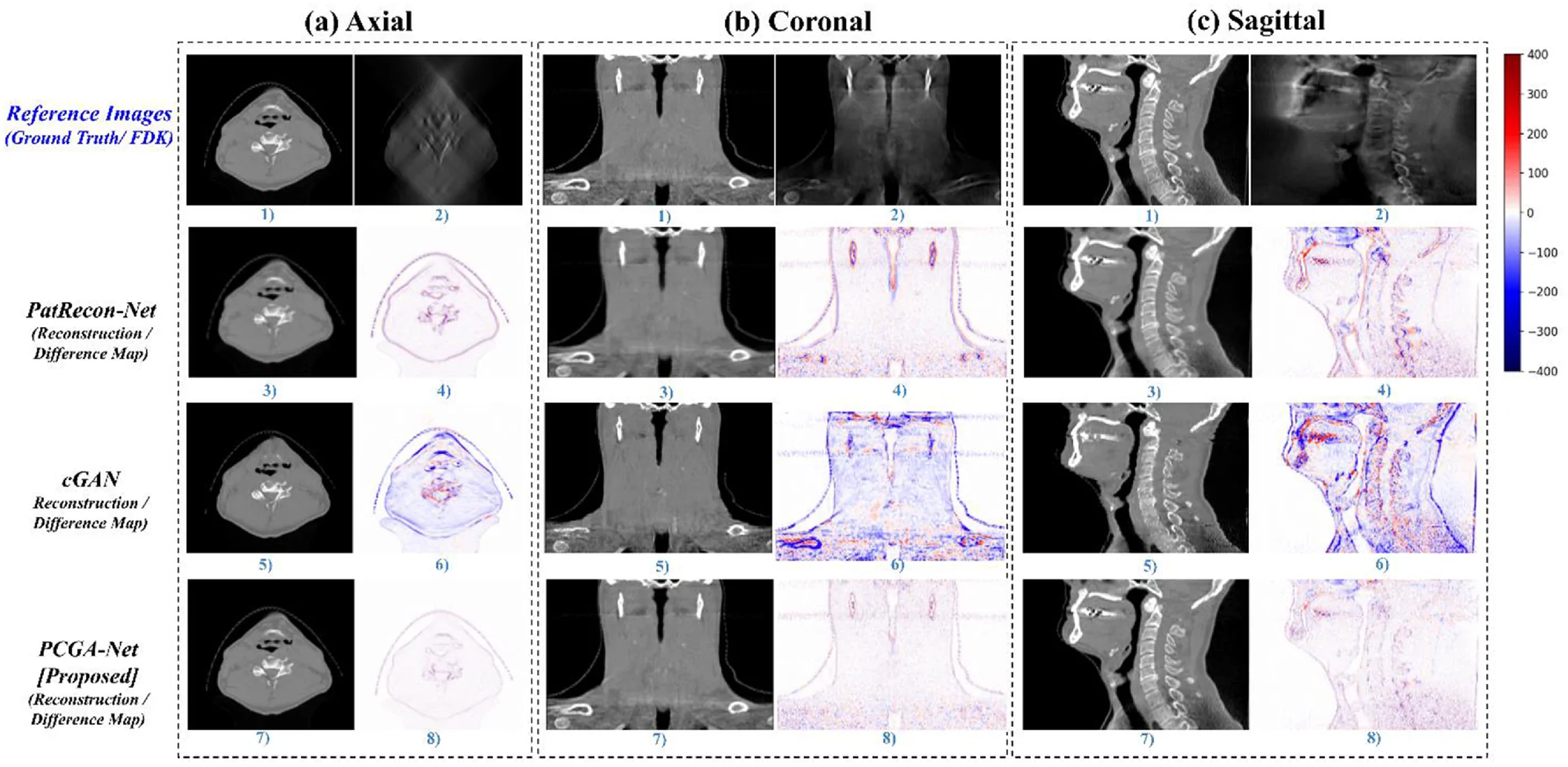
Qualitative comparison of limited-angle CBCT reconstruction methods on a representative head-and-neck case. Columns (a)–(c) show the axial, coronal, and sagittal views, respectively. In each column, the first row provides the reference images, where subpanels 1 show the ground-truth CBCT and subpanels 2 show the corresponding 90° limited-angle FDK reconstructions. The remaining rows correspond to PatRecon-Net, cGAN, and the proposed PCGA-Net, respectively; for these methods, subpanels 3, 5, and 7 show the reconstructed images, and subpanels 4, 6, and 8 show the corresponding difference maps with respect to the ground-truth CBCT. Difference maps are displayed using the color scale on the right, with intensity differences ranging from −400 to 400 HU. Compared with the 90° limited-angle FDK reconstruction and the learning-based baselines, PCGA-Net shows reduced residual artifacts and improved anatomical agreement with the ground-truth CBCT across all three anatomical planes.

Among the learning-based baselines, PatRecon-Net reduces part of the large-scale artifact pattern but still leaves visible residual errors around the body contour, lower neck soft tissue, and vertebral structures. cGAN shows more pronounced intensity bias and spatial inconsistency, with broader high-magnitude residuals distributed throughout both superficial and deep anatomical regions. In contrast, PCGA-Net achieves the closest visual agreement to the reference CBCT across all three views. The reconstructed anatomy appears more spatially coherent, with improved delineation of the cervical vertebrae, airway contour, and surrounding soft-tissue interfaces. This advantage is consistently reflected in the error maps, where PCGA-Net shows the weakest residual deviations relative to both FDK and the compared learning-based methods.

The qualitative improvement is particularly relevant in the H&N setting because this site contains dense bone-air-soft-tissue interfaces, fine anatomical structures, and immobilization-related geometric complexity, all of which make limited-angle reconstruction especially challenging. In the sagittal view, PCGA-Net better preserves the continuity of the cervical spine and adjacent soft-tissue profile, while in the coronal view it more effectively suppresses broad shading artifacts across the neck cross-section. Overall, these results indicate that PCGA-Net provides more effective artifact suppression and more faithful recovery of H&N anatomy than conventional analytic reconstruction and the compared deep-learning baselines.

#### 4.1.3. Qualitative assessment of the abdominal liver 4DCT

Figure 5 presents a representative visual comparison for the abdominal liver 4DCT cohort in the axial, coronal, and sagittal planes, together with the corresponding error maps. Under limited-angle acquisition, the FDK reconstruction shows substantial degradation throughout the upper abdomen, including broad shading, reduced soft-tissue contrast, and depth-direction blurring. These artifacts are especially evident in the coronal and sagittal views, where the extended cranio-caudal anatomy and incomplete angular coverage lead to noticeable structural distortion along the liver dome, posterior body contour, and paraspinal region.

**Figure 5.**
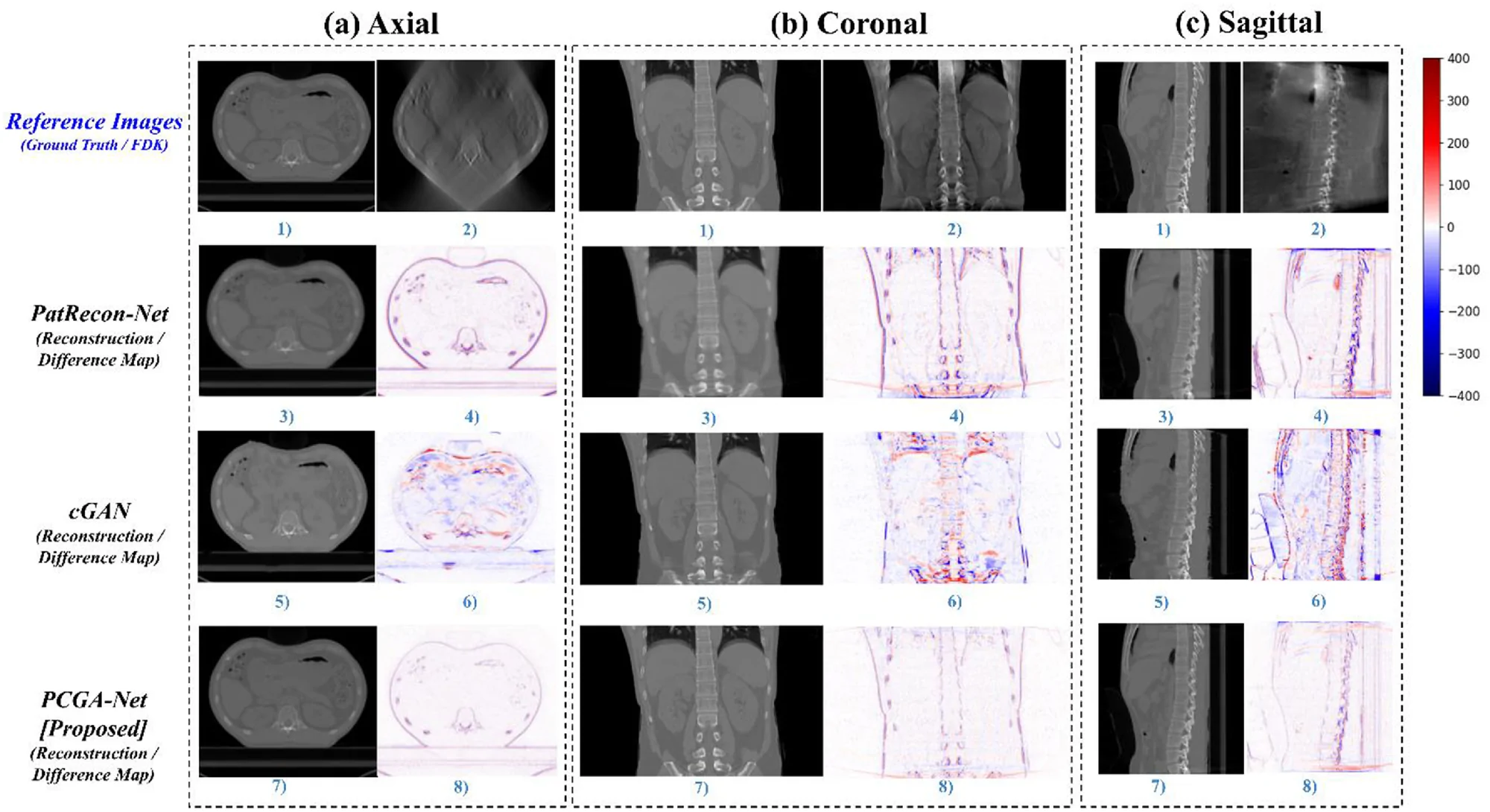
Qualitative comparison of limited-angle CBCT reconstruction methods on a representative abdominal liver 4DCT case. Columns (a)–(c) show the axial, coronal, and sagittal views, respectively. In each column, the first row provides the reference images, where subpanels 1 show the ground-truth CBCT and subpanels 2 show the corresponding 90° limited-angle FDK reconstructions. The remaining rows correspond to PatRecon-Net, cGAN, and the proposed PCGA-Net, respectively; for these methods, subpanels 3, 5, and 7 show the reconstructed images, and subpanels 4, 6, and 8 show the corresponding difference maps with respect to the ground-truth CBCT. Difference maps are displayed using the color scale on the right, with intensity differences ranging from −400 to 400 HU. Compared with the 90° limited-angle FDK reconstruction and the learning-based baselines, PCGA-Net shows reduced residual artifacts and improved anatomical agreement with the ground-truth CBCT across all three anatomical planes.

Among the compared learning-based methods, PatRecon-Net reduces some of the large-scale artifact pattern relative to FDK and preserves the global body outline reasonably well, but residual errors remain visible around the liver boundary, abdominal wall, and vertebral structures. cGAN shows more substantial intensity deviation and less stable anatomical recovery, with broader residual patterns distributed across both the abdominal soft tissue and posterior structures. In contrast, PCGA-Net provides the closest visual agreement to the reference CBCT across all three views. The reconstructed liver contour is more spatially coherent, the vertebral column is better preserved, and the surrounding abdominal soft tissue appears less affected by limited-angle shading and streaking. These improvements are consistently supported by the error maps, where PCGA-Net shows the weakest residual deviations relative to both FDK and the learning-based baselines.

The qualitative advantage of PCGA-Net is particularly relevant in the abdominal setting, where low soft-tissue contrast and respiration-associated anatomical variation make limited-angle reconstruction especially challenging. In this cohort, successful reconstruction requires not only suppression of global artifact patterns but also preservation of weak organ boundaries and large-scale anatomical continuity. Overall, the results indicate that PCGA-Net achieves more effective artifact suppression and more faithful recovery of abdominal anatomy than conventional FDK reconstruction and the compared deep-learning baselines.

### 4.2. Quantitative results

Figure 6 summarizes the overall quantitative performance of all methods across the three datasets, while the detailed numerical results for each cohort are reported in the corresponding tables below. A consistent trend can be observed across the GYN HDR brachytherapy CT, abdominal liver 4DCT, and H&N CBCT cohorts: PCGA-Net achieved the lowest MAE and the highest SSIM and PSNR in all three settings, indicating superior voxel-level accuracy, structural preservation, and overall reconstruction fidelity under limited-angle acquisition. In contrast, the conventional FDK baseline showed substantial performance degradation across all datasets, and although the learning-based baselines improved upon FDK, they remained consistently inferior to PCGA-Net. This cross-cohort pattern suggests that the proposed geometry-integrated dual-domain framework provides not only site-specific improvement, but also stable quantitative benefit across anatomically and clinically diverse limited-angle reconstruction tasks.

**Figure 6.**
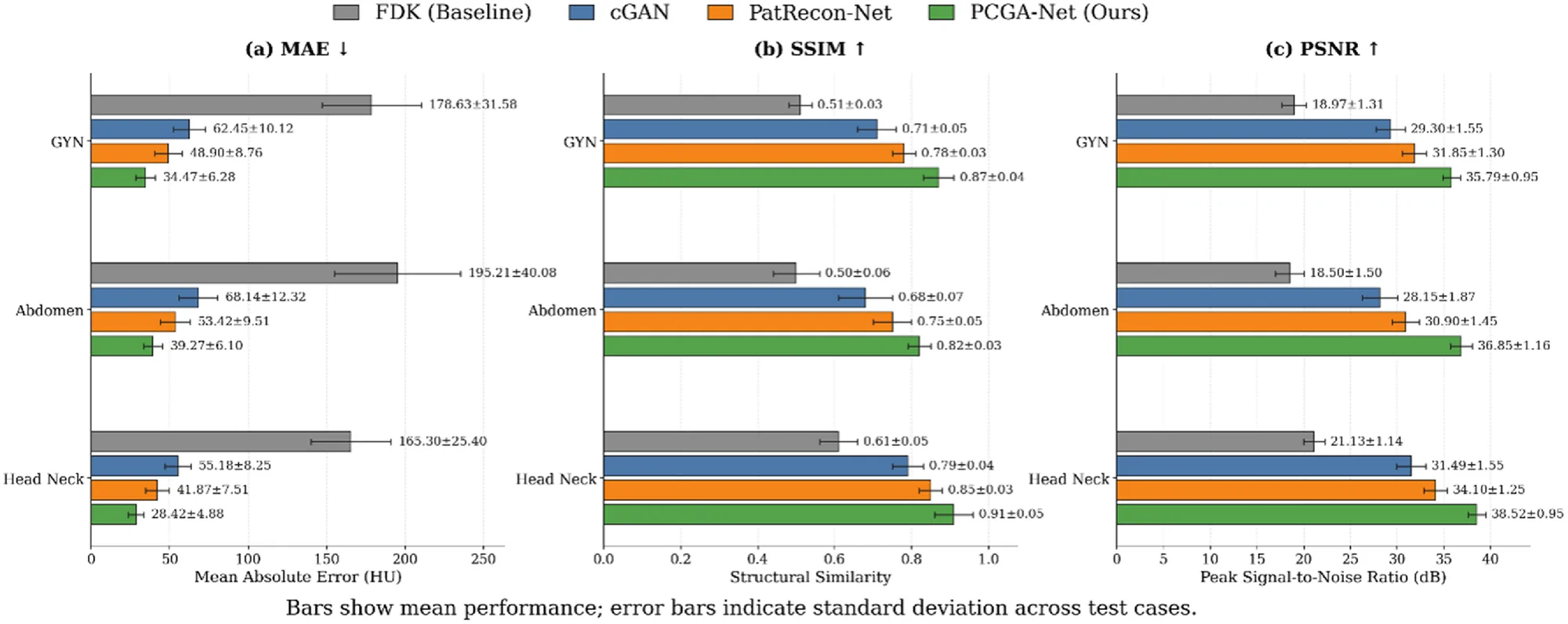
Summary of quantitative reconstruction performance across the GYN HDR brachytherapy CT, abdominal liver 4DCT, and head-and-neck CBCT cohorts. Panels (a)–(c) show MAE, SSIM, and PSNR, respectively. Bars represent mean values across the test cases, and error bars indicate standard deviation. Lower MAE and higher SSIM/PSNR indicate better reconstruction performance. Detailed numerical results and statistical comparisons are provided in the corresponding tables below.

To assess whether the observed performance differences were statistically significant, pairwise comparisons between PCGA-Net and each baseline method were performed using a two-sided paired Student’s *t*-test on the test cases within each cohort. A significance threshold of p<0.05 was adopted throughout the study. A paired test was used because all reconstruction methods were evaluated on the same set of test fractions/phases, enabling direct case-wise comparison of MAE, SSIM, and PSNR. Statistical significance is indicated by * in the corresponding tables.

#### 4.2.1. Quantitative assessment of the GYN HDR brachytherapy CT

The quantitative results for the GYN HDR brachytherapy cohort further confirm the advantage of PCGA-Net over both analytic reconstruction and the compared learning-based baselines. As summarized in table 2, PCGA-Net achieved the best performance across all three metrics, with an MAE of 34.47 ± 6.28 HU, an SSIM of 0.87 ± 0.04, and a PSNR of 35.79 ± 0.95 dB. In comparison, the conventional FDK reconstruction yielded substantially poorer accuracy (178.63 ± 31.58 HU MAE, 0.51 ± 0.03 SSIM, and 18.97 ± 1.31 dB PSNR), indicating that severe limited-angle degradation cannot be adequately resolved by analytic reconstruction alone.

Relative to FDK, PCGA-Net reduced MAE by approximately 80.7%, increased SSIM by 0.36, and improved PSNR by 16.82 dB. These gains indicate that the proposed framework substantially improves both voxel-level fidelity and structural preservation in the pelvic setting. Compared with the learning-based baselines, PCGA-Net also maintained a clear quantitative margin. Relative to cGAN, PCGA-Net reduced MAE from 62.45 ± 10.12 HU to 34.47 ± 6.28 HU, corresponding to an error reduction of about 44.8%, while improving SSIM from 0.71 ± 0.05–0.87 ± 0.04 and PSNR from 29.30 ± 1.55 dB to 35.79 ± 0.95 dB. Compared with PatRecon-Net, PCGA-Net achieved an additional 29.5% reduction in MAE, together with an absolute SSIM gain of 0.09 and a PSNR gain of 3.94 dB.

An additional point of interest is the reduction in performance variability. PCGA-Net not only achieved the best mean values, but also showed relatively small standard deviations across the test set, suggesting more stable reconstruction behavior across fractions. This is particularly relevant in the GYN HDR brachytherapy setting, where anatomical appearance may vary across treatment sessions due to applicator placement, organ filling, and local pelvic deformation. From a quantitative perspective, these results indicate that PCGA-Net improves not only average reconstruction accuracy, but also robustness under clinically realistic inter-fraction variability.

#### 4.2.2. Quantitative assessment of the H&N CBCT

The quantitative results on the H&N CBCT cohort show a consistent advantage of PCGA-Net over both conventional analytic reconstruction and the compared learning-based baselines. As summarized in table 3, PCGA-Net achieved the best performance across all evaluated metrics, with an MAE of 28.42 ± 4.88 HU, an SSIM of 0.91 ± 0.05, and a PSNR of 38.52 ± 0.95 dB. In contrast, the FDK baseline showed substantially inferior reconstruction accuracy, yielding an MAE of 165.30 ± 25.40 HU, an SSIM of 0.61 ± 0.05, and a PSNR of 21.13 ± 1.14 dB. These results indicate that conventional limited-angle reconstruction is insufficient to preserve the fine anatomical detail and structural consistency required in the H&N setting.

Relative to FDK, PCGA-Net reduced MAE by approximately 82.8%, increased SSIM by 0.30, and improved PSNR by 17.39 dB. This magnitude of improvement is particularly notable given the structural complexity of the H&N region, where dense osseous anatomy, air cavities, and soft-tissue interfaces make limited-angle reconstruction especially challenging. Compared with cGAN, PCGA-Net reduced MAE from 55.18 ± 8.25 HU to 28.42 ± 4.88 HU, corresponding to an error reduction of approximately 48.5%, while also improving SSIM from 0.79 ± 0.04–0.91 ± 0.05 and PSNR from 31.49 ± 1.43 dB to 38.52 ± 0.95 dB. Compared with PatRecon-Net, PCGA-Net further reduced MAE by about 32.1%, together with an absolute SSIM gain of 0.06 and a PSNR gain of 4.42 dB.

#### 4.2.3. Quantitative assessment of the abdominal liver 4DCT

The quantitative results on the abdominal liver 4DCT cohort demonstrate that PCGA-Net also maintains a clear advantage in this anatomically and temporally challenging setting. As summarized in table 4, PCGA-Net achieved the best performance across all three metrics, with an MAE of 39.27 ± 6.10 HU, an SSIM of 0.82 ± 0.03, and a PSNR of 36.85 ± 1.16 dB. In comparison, the conventional FDK reconstruction remained substantially degraded, with an MAE of 195.21 ± 40.08 HU, an SSIM of 0.50 ± 0.06, and a PSNR of 18.50 ± 1.50 dB. These results indicate that the incomplete angular support in abdominal imaging cannot be adequately compensated by analytic reconstruction alone, particularly in the presence of low soft-tissue contrast and respiration-related anatomical variation.

Relative to FDK, PCGA-Net reduced MAE by approximately 79.9%, increased SSIM by 0.32, and improved PSNR by 18.35 dB. Compared with cGAN, PCGA-Net reduced MAE from 68.14 ± 12.32 HU to 39.27 ± 6.10 HU, corresponding to an error reduction of about 42.4%, while also improving SSIM from 0.68 ± 0.07–0.82 ± 0.03 and PSNR from 28.15 ± 1.87 dB to 36.85 ± 1.16 dB. Compared with PatRecon-Net, PCGA-Net achieved an additional 27.4% reduction in MAE, together with an absolute SSIM gain of 0.07 and a PSNR gain of 5.95 dB. These margins are quantitatively meaningful because the abdominal cohort is intrinsically more difficult than the pelvic and H&N cohorts, owing to weaker organ contrast and more pronounced deformation across respiratory phases.

### 4.3. Population-only comparison and patient-specific adaptation

To separate the reconstruction capability of the population-pretrained model from the contribution of longitudinal patient-specific priors, we performed two complementary adaptation analyses. First, table 5 separates the population-only (N=0) and fully adapted configurations for cGAN, PatRecon-Net, and PCGA-Net in each cohort. Within each configuration, the learned methods use the same patient-information constraints. The fully adapted means reproduce the corresponding results in tables 2–4, while FDK remains the non-adaptable analytic reference reported in those tables. The fully adapted configurations used 2 prior fractions for the GYN HDR brachytherapy cohort, 10 prior fractions for the H&N CBCT cohort, and 3 prior fractions/phases for the abdominal liver 4DCT cohort. The later evaluation fractions/phases remained held out at all adaptation levels, and the acquisition geometry, preprocessing, and evaluation procedures were unchanged.

Under population-only inference, PCGA-Net achieved MAEs of 58.06, 47.88, and 82.66 HU in the GYN, H&N, and liver cohorts, respectively. The corresponding values were 85.0, 76.0, and 112.0 HU for cGAN and 70.0, 62.0, and 96.0 HU for PatRecon-Net. PCGA-Net also yielded higher SSIM and PSNR in each cohort: 0.79 and 33.29 dB for GYN, 0.80 and 33.37 dB for H&N, and 0.71 and 30.75 dB for liver. Thus, the population-only comparison evaluates the reconstruction pipelines without incorporating target-patient adaptation data.

Patient-specific adaptation improved all three metrics for every learned method. For cGAN, MAE decreased by 26.53%, 27.39%, and 39.16% in the GYN, H&N, and liver cohorts, respectively; the corresponding reductions for PatRecon-Net were 30.14%, 32.47%, and 44.35%. Under the fully adapted protocol, PCGA-Net retained lower MAE and higher SSIM and PSNR than both learned baselines in all three cohorts. The two sets of comparisons therefore distinguish differences among complete reconstruction pipelines under matched patient-information conditions from the additional benefit obtained by adapting each model to the target patient. They do not isolate the contribution of any individual architectural module.

As shown in table 5, population-only PCGA-Net retained meaningful reconstruction capability in all three cohorts, and patient-specific adaptation improved every evaluated metric for PCGA-Net. The relative MAE reductions were 40.63%, 40.64%, and 52.49% for the GYN HDR, H&N, and liver cohorts, respectively. The corresponding relative SSIM increases were 10.13%, 13.75%, and 15.49%, and the absolute PSNR increases were 2.50, 5.15, and 6.10 dB, respectively. Thus, the site-specific population model provides a feasible no-adaptation operating point, whereas longitudinal patient-specific adaptation supplies additional anatomical information that materially improves reconstruction accuracy.

To examine how reconstruction changes with the amount of patient-specific information, we further performed an adaptation-dose ablation in the H&N cohort, which provided 10 prior fractions and therefore supported a multi-level longitudinal analysis. The model was adapted using N=0,2,4,6,8, or 10 prior fractions, while the same later fractions were retained for evaluation at every setting.

The H&N adaptation-dose results show an overall improvement as more longitudinal information was introduced. Relative to N=0, using 2 prior fractions reduced MAE by 17.50% and increased SSIM by 5.00%. At N=10, MAE was reduced by 40.64% and SSIM was increased by 13.75%. The improvement was not strictly monotonic for every metric: MAE increased slightly from 39.50 HU at N=2 to 39.96 HU at N=4, while SSIM increased from 0.84 to 0.85. Performance subsequently improved at N=6, 8, and 10. This behavior suggests that patient-specific adaptation is beneficial overall, but the marginal gain from each additional fraction is nonuniform and may depend on the anatomical information represented by the available prior images.

## 5. Discussion

This study addresses a clinically important but methodologically difficult reconstruction setting: recovering volumetric CBCT from continuous limited-angle acquisition. In this regime, the challenge is not simply the presence of visible streaking or shading artifacts, but the fact that incomplete angular sampling removes part of the information required for stable volumetric recovery. This makes limited-angle CBCT fundamentally different from routine image denoising or post-processing tasks. The findings of this study suggest that improved reconstruction in this setting depends on respecting where the information loss originates. Because the incompleteness is introduced in projection space but manifests clinically in image space, an effective solution must coordinate both domains rather than relying on image restoration alone. This is the main rationale for combining projection completion, analytic geometric transformation, and image-domain refinement within a single framework.

One important implication of the current results is that explicit imaging geometry remains essential even in the era of generative reconstruction. Diffusion-based models provide strong generative capacity and can recover fine anatomical detail more effectively than conventional restoration networks, but such capacity is most useful when the generation process is constrained by physically meaningful mappings. In limited-angle reconstruction, unconstrained image synthesis can easily improve appearance without guaranteeing compatibility with the measured rays. By embedding the GARM between projection completion and volumetric refinement, the present framework uses scanner geometry not merely as implementation detail, but as a structural constraint on what the model is allowed to generate. This point is important for future research as well: for short-arc CBCT, the central question is not how to generate the sharpest possible image, but how to generate a volumetric image whose recovered structures remain accountable to the acquisition geometry.

The cGAN comparison should be interpreted as a pipeline-level comparison with a representative image-domain conditional generative method rather than an architecture-matched ablation. cGAN performs limited-angle artifact correction after FDK reconstruction and does not include projection completion, the analytic GARM, or projection-image cycle supervision. Adding these components would convert cGAN into a different dual-domain reconstruction framework and would no longer preserve the original pix2pix design. Accordingly, the observed differences reflect the combined effect of the reconstruction formulation and model class: image-only post-reconstruction correction for cGAN versus projection-geometry-image coupling for PCGA-Net.

PatRecon-Net is consequently a valid few-view projection-to-volume comparator, but it remains a single-stage and single-domain method. The comparison preserves the baseline’s public input interface and original reconstruction principle, while the proposed method evaluates a different hypothesis: that explicit projection completion and geometry-mediated cross-domain coupling improve limited-angle reconstruction. The quantitative comparison should therefore be interpreted at the complete-pipeline level and not as an isolated assessment of network capacity.

The multi-site evaluation further suggests that limited-angle reconstruction should not be viewed as a site-specific engineering problem. Although all three cohorts share the same broad reconstruction objective, the actual difficulty differs substantially by anatomy and workflow. In GYN HDR brachytherapy, the main challenge lies in preserving pelvic soft tissue and device-related geometry in a crowded procedural environment. In the abdominal liver cohort, low soft-tissue contrast and respiration-associated anatomical variation make the boundary between true organ structure and limited-angle artifact particularly ambiguous. In the H&N cohort, dense osseous-air-soft-tissue interfaces and elongated anatomy amplify direction-dependent distortion. That the same reconstruction principle remains effective across these settings indicates that geometry-aware cross-domain modeling captures a transferable part of the inverse problem, while the patient-specific adaptation stage remains necessary to absorb anatomy-dependent appearance variation. This has practical significance, because future deployment of limited-angle CBCT will likely require both globally learned geometric priors and local adaptation to patient-specific imaging context.

The current framework also has an important clinical interpretation. It should be understood as a reconstruction strategy for situations in which full-angle CBCT is difficult, undesirable, or operationally incompatible with the workflow, rather than as a universal replacement for standard full-scan CBCT. In radiotherapy and image-guided procedures, limited-angle acquisition becomes relevant precisely because it may reduce scan burden, shorten interruption of treatment or intervention, and better fit constrained procedural environments. In such scenarios, the goal is often not to reproduce every aspect of a diagnostic-quality full-angle CBCT, but to recover sufficient volumetric information for anatomy-aware guidance, treatment adaptation, or procedural verification. This perspective is important when judging model performance: the value of short-arc reconstruction lies in whether it restores clinically usable three-dimensional structure under realistic acquisition constraints.

The primary comparisons in tables 2–4 represent the fully adapted operating point: cGAN, PatRecon-Net, and PCGA-Net used the same target-patient prior fractions/phases and adaptation budget. Table 5 separately reports population-only and fully adapted configurations, while table 6 characterizes the effect of the number of prior fractions for PCGA-Net in the H&N cohort. These analyses distinguish reconstruction without target-patient adaptation from reconstruction supported by same-patient longitudinal information. Generalizability remains limited to the evaluated cohorts, site-specific population pretraining, and specified acquisition and adaptation protocols.

**Table 6.** Head-and-neck adaptation-dose ablation as a function of the number of target-patient prior fractions used for fine-tuning. N=0 denotes population-only inference and N=10 denotes the fully adapted configuration. Values in parentheses are relative changes from N=0, rather than changes from the immediately preceding column. Lower MAE and higher SSIM indicate better reconstruction performance.

Metric	N=0 Population-only	N=2	N=4	N=6	N=8	N=10 Fully adapted
MAE (HU) ↓	47.88	39.50 (−17.50%)	39.96 (−16.54%)	36.00 (−24.81%)	34.25 (−28.47%)	28.42 (−40.64%)
SSIM ↑	0.80	0.84 (+5.00%)	0.85 (+6.25%)	0.87 (+8.75%)	0.88 (+10.00%)	0.91 (+13.75%)

The adaptation analyses clarify the intended clinical interpretation of PCGA-Net. The population-only results provide a no-adaptation operating point relevant to first-fraction imaging or to a patient for whom no longitudinal prior is available, whereas the fully adapted results represent repeated image-guided or adaptive workflows in which earlier fractions can be used to establish a stronger patient-specific prior. Across all three cohorts, the population-pretrained model remained functional without target-patient fine-tuning, but full adaptation produced substantial additional improvements. The H&N adaptation-dose experiment further showed an overall benefit from increasing the number of prior fractions, although the marginal improvement was not strictly monotonic. These findings confirm that the primary adapted results reflect both the reconstruction capability of the population model and the additional information supplied by same-patient longitudinal imaging; they should not be interpreted as patient-independent zero-shot performance.

The detailed adaptation-dose curve was limited to the H&N cohort because it provided 10 longitudinal prior fractions, whereas the GYN HDR and liver cohorts provided only 2 and 3 adaptation fractions/phases, respectively. Those smaller adaptation sets permit comparison of the population-only and fully adapted endpoints, as reported in table 5, but do not provide a similarly resolved range for characterizing an adaptation-dose relationship. Moreover, each adaptation level requires separate optimization and evaluation of two three-dimensional diffusion branches coupled through differentiable cone-beam geometry, creating a substantial computational burden. The present study therefore combines a three-cohort endpoint analysis with a detailed H&N dose–response analysis. Future work should extend the multi-level ablation to additional anatomical sites, evaluate alternative selections and orderings of prior fractions, and determine whether a smaller adaptation set can achieve a clinically favorable balance between reconstruction accuracy and computational cost.

Several limitations remain and provide clear directions for future work. First, the evaluated cohorts rely on CT- or 4DCT-based projection simulation, which is appropriate for controlled development because it provides artifact-reduced reference anatomy and precisely paired projection-volume data, but it does not fully reproduce the scatter, detector effects, and system-specific degradation observed in real CBCT acquisition. Broader validation on raw clinical projection data will therefore be necessary. Second, the present implementation assumes a fixed acquisition geometry and a predefined short-arc configuration. In future studies, it will be important to test robustness to variable arc lengths, angular placement, acquisition protocols, and noise conditions, since these factors are likely to differ across scanners and clinical workflows. Third, although the current model generalizes across multiple anatomical sites, its adaptation strategy remains cohort-specific. A meaningful next step would be to investigate whether a more unified foundation-style reconstruction model can maintain cross-site generality while still supporting efficient patient-level refinement. Finally, the evaluation in future work should move beyond image similarity alone toward task-driven endpoints, such as contouring stability, registration accuracy, dose recalculation, or device localization. These endpoints would better determine whether improved image reconstruction translates into improved image-guided decision-making.

A further limitation is that the fixed loss weights in equation (9) were not subjected to an exhaustive grid search. Repeated joint training of two three-dimensional diffusion branches with differentiable cone-beam operators is computationally intensive; therefore, the current empirical coefficients should not be interpreted as globally optimal. Future studies will investigate adaptive loss-weighting strategies, such as uncertainty-based or gradient-normalized balancing, and will evaluate the sensitivity of reconstruction accuracy and geometric consistency to these coefficients.

A further limitation concerns the provenance of the H&N projection data. The H&N source volumes were reconstructed clinical CBCT images that were subsequently forward-projected using the same nominal cone-beam geometry employed by the GARM. In strict inverse-problem terminology, this matched re-projection/reconstruction design constitutes an inverse crime and may yield more favorable data consistency than would be obtained from independent raw detector measurements. It does not capture acquisition-model mismatch, scatter, detector lag, calibration errors, truncation, beam-hardening effects, or the native projection-noise distribution. Accordingly, the H&N results should be interpreted as controlled reconstruction performance on clinically derived anatomy rather than as validation on raw clinical CBCT projections. Prospective evaluation using independently acquired raw projection data and scanner-specific corrections is required before clinical deployment.

The current experiments also use idealized projection measurements without an explicit photon-statistics or electronic-noise model. This allows the effect of missing angular coverage to be studied in isolation, but it does not reproduce the strong coupling between limited-angle ill-conditioning and measurement noise encountered in low-dose clinical CBCT. Consequently, the reported results may overestimate performance under noisy raw-projection conditions. Future studies will evaluate multiple photon-flux levels, Poisson and electronic noise, scatter and beam-hardening effects, detector lag, and geometry/calibration mismatch, followed by validation on independently acquired raw clinical projections.

Overall, this study supports a broader view of limited-angle CBCT reconstruction as a geometry-constrained inverse problem that is modulated by anatomy, workflow, and acquisition context. Within this setting, neither purely analytic reconstruction nor unconstrained generative modeling is sufficient by itself. The practical value of the proposed framework lies in showing that projection-domain completion, analytic geometric consistency, and image-domain refinement can be organized into a coherent reconstruction strategy for short-arc CBCT. More broadly, the study suggests that future progress in this area will depend less on increasing model complexity alone and more on designing reconstruction methods in which learned priors and imaging physics remain tightly coupled throughout the reconstruction process.

## 6. Conclusion

In conclusion, this study presents PCGA-Net, a physics-constrained and geometry-aware reconstruction framework for limited-angle CBCT that jointly performs projection completion, analytic geometric reconstruction, and volumetric refinement within a unified cross-domain design. By explicitly coupling learned generative priors with cone-beam imaging geometry, the proposed method addresses the intrinsic ill-posedness of short-arc reconstruction more effectively than conventional analytic reconstruction and the compared learning-based baselines. The expanded evaluation across GYN HDR brachytherapy CT, abdominal liver 4DCT, and H&N CBCT further indicates that the framework is robust to substantial variation in anatomy, motion pattern, and clinical workflow. These findings support PCGA-Net as a promising strategy for producing anatomically faithful volumetric images from incomplete angular measurements in constrained image-guided therapeutic settings.

The primary learned-method comparisons used matched patient-specific adaptation conditions. The population-only and fully adapted configurations are reported separately in table 5, and the H&N longitudinal analysis in table 6 quantifies the dependence on the number of prior fractions. These results distinguish the reconstruction capability of the population-pretrained model from the additional benefit of patient-specific information. Validation on independent raw projection data and broader acquisition settings remains necessary.

## Data Availability

The data cannot be made publicly available upon publication because they contain sensitive personal information. The data that support the findings of this study are available upon reasonable request from the authors. Network architecture details available at https://doi.org/10.1088/1361-6560/aeb59b/data1.
